# The Efficacy of a Brief, Altruism-Eliciting Video Intervention in Enhancing COVID-19 Vaccination Intentions Among a Population-Based Sample of Younger Adults: Randomized Controlled Trial

**DOI:** 10.2196/37328

**Published:** 2022-05-30

**Authors:** Patricia Zhu, Ovidiu Tatar, Gabrielle Griffin-Mathieu, Samara Perez, Ben Haward, Gregory Zimet, Matthew Tunis, Ève Dubé, Zeev Rosberger

**Affiliations:** 1 Lady Davis Institute for Medical Research Jewish General Hospital Montreal, QC Canada; 2 Department of Psychiatry McGill University Montreal, QC Canada; 3 Research Center Centre Hospitalier de l’Université de Montréal Montreal, QC Canada; 4 Cedars Cancer Center McGill University Health Center Montreal, QC Canada; 5 Department of Oncology McGill University Montreal, QC Canada; 6 Division of Adolescent Medicine Indiana University School of Medicine Indianapolis, IN United States; 7 National Advisory Committee on Immunization Secretariat Centre for Immunization Readiness Public Health Agency of Canada Ottawa, ON Canada; 8 Faculty of Social Sciences; Anthropology University of Laval Québec, QC Canada; 9 Department of Psychology McGill University Montreal, QC Canada

**Keywords:** COVID-19, vaccination, altruism, prosocial motives, video intervention, randomized controlled trial, younger adults, vaccine hesitancy, public health, youth, digital intervention, health intervention, health promotion, web survey, digital health, online health, health information

## Abstract

**Background:**

High COVID-19 vaccine uptake is crucial to containing the pandemic and reducing hospitalizations and deaths. Younger adults (aged 20-39 years) have demonstrated lower levels of vaccine uptake compared to older adults, while being more likely to transmit the virus due to a higher number of social contacts. Consequently, this age group has been identified by public health authorities as a key target for vaccine uptake. Previous research has demonstrated that altruistic messaging and motivation is associated with vaccine acceptance.

**Objective:**

This study had 2 objectives: (1) to evaluate the within-group efficacy of an altruism-eliciting short, animated video intervention in increasing COVID-19 vaccination intentions amongst unvaccinated Canadian younger adults and (2) to examine the video’s efficacy compared to a text-based intervention focused exclusively on non-vaccine-related COVID-19 preventive health measures.

**Methods:**

Using a web-based survey in a pre-post randomized control trial (RCT) design, we recruited Canadians aged 20-39 years who were not yet vaccinated against COVID-19 and randomized them in a 1:1 ratio to receive either the video intervention or an active text control. The video intervention was developed by our team in collaboration with a digital media company. The measurement of COVID-19 vaccination intentions before and after completing their assigned intervention was informed by the multistage Precaution Adoption Process Model (PAPM). The McNemar chi-square test was performed to evaluate within-group changes of vaccine intentions. Exact tests of symmetry using pairwise McNemar tests were applied to evaluate changes in multistaged intentions. Between-group vaccine intentions were assessed using the Pearson chi-square test postintervention.

**Results:**

Analyses were performed on 1373 participants (n=686, 50%, in the video arm, n=687, 50%, in the text arm). Within-group results for the video intervention arm showed that there was a significant change in the intention to receive the vaccine (*χ*^2^_1_=20.55, *P*<.001). The between-group difference in postintervention intentions (*χ*^2^_3_=1.70, *P*=.64) was not significant. When administered the video intervention, we found that participants who had not thought about or were undecided about receiving a COVID-19 vaccine were more amenable to change than participants who had already decided not to vaccinate.

**Conclusions:**

Although the video intervention was limited in its effect on those who had firmly decided not to vaccinate, our study demonstrates that prosocial and altruistic messages could increase COVID-19 vaccine uptake, especially when targeted to younger adults who are undecided or unengaged regarding vaccination. This might indicate that altruistic messaging provides a “push” for those who are tentative toward, or removed from, the decision to receive the vaccine. The results of our study could also be applied to more current COVID-19 vaccination recommendations (eg, booster shots) and for other vaccine-preventable diseases.

**Trial Registration:**

ClinicalTrials.gov NCT04960228; https://clinicaltrials.gov/ct2/show/NCT04960228

## Introduction

SARS-CoV-2 has caused the greatest pandemic of our lifetime. At the time of writing, the virus had infected 251 million people and killed over 5 million worldwide [1]. To contain the COVID-19 pandemic, governments have recommended and mandated preventive health measures, such as physical distancing, mask wearing, and restrictions on indoor and outdoor gatherings. Although these measures have been instrumental in reducing virus transmission and the burden on the health care system, they have also had severe impacts on the economy and individual well-being [2-4].

Following a rapid mobilization and development process, COVID-19 vaccination was introduced in late 2020, and widespread vaccination has since been encouraged for the general population. In Canada, vaccinating against COVID-19 has likely saved 476,000 lives [5]. Compared to those who are vaccinated, unvaccinated individuals make up a disproportionally higher percentage of infection cases (61.9% vs 38.1%), hospitalizations (77.3% vs 22.7%), and deaths (74.6% vs 25.4%) [6]. Further, there is evidence that vaccination has helped reduce virus transmission [7].

Vaccine hesitancy, which refers to a set of attitudes and beliefs that may lead to delay or refusal of 1 or more vaccines despite their availability [8,9], poses a significant threat to achieving sufficient COVID-19 vaccination rates to mitigate the pandemic. Younger age has been associated with vaccine hesitancy [10-14]. Additionally, younger adults often experience mild or asymptomatic infections [15,16] and are more socially active. In Canada, this age group also demonstrates lower adherence to other preventive health measures (eg, social distancing) [17,18]. Thus, younger adults play an important role in virus transmission. To protect the Canadian population at large, it is important to ensure adequate vaccine uptake amongst younger adults.

Although providing basic vaccine education to the population is critical, research has shown that correcting vaccine misinformation and refuting vaccine myths are largely ineffective in enhancing vaccine intentions [19]. This resistance may be attributable in part to confirmation bias. Studies have shown that vaccine-hesitant individuals are less receptive to new information that disconfirms their beliefs [19,20]. Additionally, vaccine hesitancy cannot be understood as a total refusal or acceptance of vaccination but rather as a continuum. Individuals in different stages of vaccine decision-making have different attitudes and beliefs toward vaccination [21,22]. Therefore, the efficacy of interventions designed to address vaccine hesitancy might be moderated by the set of attitudes, beliefs, and cognitions a specific individual has toward vaccination.

A novel and promising approach is to develop interventions that elicit altruism, that is, intentional and voluntary action in which the primary goal is to increase the welfare of another person [23,24]. Previous hypothetical and laboratory game studies have found that altruistic messages can increase vaccination intentions [25-27] or demonstrated that altruistic motives were related to self-reports of actual vaccine intentions or behaviors. However, few studies have experimentally elicited altruism to examine its impact on vaccine intentions [28,29], and none have used a video-based intervention. Younger adults have lower concerns of hospitalization and mortality than older adults [30] and thus may perceive receiving a COVID-19 vaccine as less personally beneficial. To increase vaccination intentions and uptake amongst this age group, it could be more effective to highlight messages of altruism and the protection of others rather than oneself [12,31].

Considering the need to address hesitancy toward COVID-19 vaccination amongst younger adults, the aim of this study was to evaluate the efficacy of a short video intervention eliciting altruistic motives for vaccination. Understanding the effectiveness of altruism-based messaging could inform public health communications targeting COVID-19 vaccine uptake in this age group. The specific objectives were to estimate (1) pre- to postintervention change of COVID-19 vaccine intentions and (2) between-group COVID-19 vaccine intentions postintervention.

## Methods

### Trial Design

We used a 2-arm parallel randomized pre-post design. Participants in a web-based survey were randomly allocated in a 1:1 ratio to the video-based intervention or the active control arm consisting of a text-based intervention. The study was designed to detect a significant pre-post increase in COVID-19 vaccine intentions in the video intervention group and the superiority of the video intervention compared to the text intervention in eliciting pro-COVID-19 vaccine intentions. We used the Consolidated Standards of Reporting Trials (CONSORT) statement to report the results [32].

### Participants and Study Setting

Participants from all Canadian provinces or territories who met following eligibility criteria were enrolled in the study: (1) not vaccinated for COVID-19, (2) age range of 20-39 years, (3) Canadian resident, and (4) willing to complete the survey in either English or French. To ensure a balanced participation in the study and informed by the Canadian Census data, we used quota sampling for the primary language spoken at home (80% Anglophones, 20% Francophones); biological sex (50% males, 50% females), annual total income before taxes of all members of the household before the pandemic (50% more than CA $75,000 [US $58,563.80], 50% less than CA $75,000), and population density (80% urban, 20% rural). During data collection (July 30-September 13, 2021), the daily incidence of COVID-19 was rising, signaling the emergence of the fourth pandemic wave in Canada that reached its peak mid-September, when about 4300 new daily cases were reported nationwide. In this period, about one-third of daily cases were reported in Canadians aged 20-39 years and the estimated daily COVID-19 incidence in this age group reached 1500 (35% of total daily cases) [33]. In Canada, our target population became eligible for COVID-19 vaccination in April-May 2021, although provincial rollout varied widely. Therefore, as of April 17, 2021, the national cumulative percentage of individuals aged 20-39 years who received at least 1 COVID-19 vaccine dose was only about 9%. Vaccine uptake increased sharply in the upcoming months and the cumulative percentage of individuals in this age group who received at least one dose reached about 62% by June 5th, 2021. During data collection, the estimated national vaccine coverage (at least 1 dose) in individuals aged 20-39 years increased from about 72% at the start to 78% [34]. In this period that corresponded with the beginning of the academic year, extensive public health interventions (eg, messages distributed through media) aiming at increasing vaccine uptake were ongoing and vaccination mandates were beginning to be implemented in some jurisdictions (eg, Quebec).

### Study Procedures

Data collection was carried out by Dynata, an international online market research company with experience in programing surveys and collecting data for universities and companies in various fields (eg, public health, politics). Dynata used a combination of recruitment methods (eg, its own website, direct emails, ads on social media) to recruit participants. At the beginning of the survey, we checked whether participants’ electronic device (the survey could be completed on a smartphone, computer, or tablet) had adequate video and sound capabilities to complete the survey. After providing electronic consent, participants deemed eligible to participate were randomly allocated to 1 of the 16 strata based on the 4 quota sampling criteria (ie, primary language, biological sex, income, and population density; see Multimedia Appendix 1 for details). Within each stratum, a random concept picker approach was used to ensure a 1:1 allocation. Correspondingly, the first participant of a pair was randomly allocated to the intervention or the control arm and the second participant to the opposite arm. If a participant did not finish the survey (incomplete data), that place in the pair was allocated to the next participant. Thus, the quota in each stratum was filled in pairs and ensured a balanced group allocation throughout the data collection period.

After randomization, participants completed the remaining baseline sociodemographic questionnaire and provided their intentions to receive a COVID-19 vaccine. Then, they participated in the intervention (watched a short video eliciting altruism motives) or read a text related to general hygiene and preventive measures (active control group). All participants were prompted that attention check questions would follow. Those who did not correctly identify the names of the video characters were offered the possibility to watch the video a second time. Those who decided to watch the video again but still answered incorrectly were terminated. The video could be paused but not skipped or muted. Participants could not continue the survey until the video had been played entirely. In the active control arm, the sequence of information sections was randomized (to control for bias attributable to presentation order) and participants could neither skip sections nor progress to the next section until 10 seconds had elapsed to encourage careful reading. After each section, participants answered an attention check question asking them to identify a measure that was not mentioned in the section they had just read. Participants who answered all 3 attention check questions incorrectly were terminated.

Immediately after completing the intervention, we reassessed their intentions to receive a COVID-19 vaccine. Subsequently, participants answered additional questions (offered after the second assessment of vaccine intentions to avoid response bias), which included flu vaccination status, health care professional status, smoking history, and measures of altruism, empathy, and psychological distress. Only participants who provided complete survey data were retained in the final database. Participants were compensated by Dynata according to the reward system in which points are earned that can be later redeemed for company rewards (eg, Amazon, Starbucks).

### Interventions

#### Video Intervention

Because mobile streaming is highly popular in our target age group [35], we decided to use a video-based intervention to maximize its acceptability and minimize study attrition. The development of the intervention was informed by a literature review conducted by our team showing that eliciting prosocial motives (altruism) can increase vaccine intentions. Accordingly, the messaging was framed around the concept of social benefit of vaccination by emphasizing the importance of indirectly protecting the health of vulnerable individuals who either cannot receive the vaccine (eg, children under the age of 5 years) or might develop an insufficient immune response (eg elderly, immunocompromised) [36-41]. Moreover, protecting children and the elderly and providing details about negative health outcomes caused by infection were found to elicit empathy and altruism and increase vaccine acceptability in young adults [28,42,43]. Because narratives represent an essential component of human communication and their use has been recommended for health behavior change interventions [44], we used this approach to emphasize the importance of receiving the vaccine for protecting others. Finally, we drew a parallel between the collective benefits of having a public health system and the social benefits of being adequately vaccinated.

The development of the intervention unfolded in following phases: First, we developed the script to focus on 3 characters with different COVID-19 vulnerability profiles (ie, John, 82 years old, vaccinated but at risk because of his age; Simon, 4 years old, not eligible for vaccination at the time of the study; and Marie, 32 years old, at risk of infection because of the immunosuppressive effects of chemotherapy). Subsequently, an initial storyboard was created by Akufen (a Montreal-based media design company), which was further refined and produced in video format. Adjustments were made based on the feedback received from 5 young adults (aged 20-39 years who had not yet received the COVID-19 vaccine) who viewed the video and participated in a focus group in June 2021. The final animated character video was 2 minutes 47 seconds in length. (Click to view the videos in English [45] or French [46]). All narration was completed by an experienced, fully bilingual professional narrator.

#### Text Intervention

Consistent with the widespread use of public health messaging campaigns during the pandemic focusing on promoting preventive health behaviors, we decided to include an active instead of a placebo control group. We developed the text-based intervention by selecting *non-vaccine-related* preventive health behavior recommendations disseminated through the Public Health Agency of Canada’s website [47]. The text-based intervention was limited to about 450 words to ensure a reading time similar to the duration of the video-based intervention. Recommendations were divided into 3 sections: travel restrictions (eg, mandatory COVID-19 testing, mandatory isolation), general hygiene (eg, handwashing, mask wearing), and physical distancing (eg, avoiding closed spaces, maintaining a physical distance of 2 m from people outside of your household). See Multimedia Appendix 2 for the text intervention and attention check questions.

### Measures

#### Baseline Sociodemographics

Baseline sociodemographics included continuous (ie, age) and categorical (province or territory, ethnicity, self-perceived visible minority [yes/no], gender identity, identification as a parent [yes/no], language spoken at home [English, French, other], postsecondary education attainment [yes/no], and income [CA $10,000 increments]) variables. Variables with a small cell count for some categories were recategorized. Provinces or territories were recategorized into Western, Central, and Eastern Canada. The 9 categories used by Statistics Canada to measure self-reported ethnic origins [48] were recategorized into North American Aboriginal, other North American (eg, Canadian, American), European, Asian, and other (ie, Caribbean, Latin, Central and South American, African, dual/mixed ethnicities, and uninterpretable open-ended responses). We used multiple validated categories [49] to measure gender identity that captures men and women’s socially constructed roles, identities, and behaviors and retained for analyses 3 categories: male, female, and gender diverse (ie, transgender male/trans man/female-to-male, transgender female/trans woman/male-to-female, genderqueer, neither exclusively male nor female, other [open ended], and prefer not to answer).

#### Main Outcome

Based on the World Health Organization (WHO) Strategic Advisory Group of Experts on Immunization (SAGE) Working Group definition, vaccine hesitancy is considered on a continuum, which implies that using a binary (yes/no) would not allow for a precise, nuanced understanding of where individuals are in their vaccination decision-making process. Therefore, to measure COVID-19 vaccine intentions, we used a stage-based model of health decision-making, the Precaution Adoption Process Model (PAPM) [50]. Informed by the PAPM, we asked participants, “Which of the following best describes your thoughts about a COVID-19 vaccine?” and allowed participants to place themselves in 1 of 4 nominal intention stages: (1) *unengaged* (“At this moment, I have not thought about receiving the COVID-19 vaccine.”), (2) *undecided* (“At this moment, I am undecided about receiving the COVID-19 vaccine.”), (3) *decided not* (“At this moment, I do NOT want to receive the COVID-19 vaccine.”), and (4) *decided to* (“At this moment, I do want to receive the COVID-19 vaccine.”).

#### Additional Measures

Additional measures included following dichotomous (yes/no) variables: identification as a caregiver for an elderly person, identification as a health care professional, receiving a COVID-19 test, influence of religion on health decisions, and seasonal influenza vaccine uptake in the past 12 months. Smoking history was captured by 3 categories: *never smoked*, *smoked in the past but not anymore*, and *currently a smoker*. Vaccination uptake of all recommended vaccines since birth was captured by 3 categories: *all vaccines*, *some vaccines*, and *no vaccines*. The validated 6-point-item (*excellent* to *very poor*) measure of self-perceived health status [51] was dichotomized into *excellent or very good* and *good or less*. Empathy was assessed using the validated 16-item Toronto Empathy Questionnaire (TEQ) [52]. Psychological distress was assessed using the validated 6-item Kessler Psychological Distress Scale (K6) [53]. Altruism was assessed using the validated 5-item altruism subscale from the Prosocial Tendencies Measure (PTM) [54].

### Sample Size

To calculate the required sample size for the within-participant change in vaccine hesitancy (ie, pre- to postintervention), we used survey data that showed that in January/February 2021, approximately 40% of Canadians aged 20-39 years were hesitant toward a COVID-19 vaccine (ie, don’t know yet or would refuse vaccination) [55,56]. Estimating a 5% decrease in hesitancy in the intervention group and a correlation of about 0.4 between paired observations, the intervention group required a sample size of 907 pairs for detecting a 5% change in marginal proportions at a power of 80% and 2-sided significance of 5% [57]. To detect a 5% superiority of the video intervention in increasing vaccine intentions compared to the active control group at a power of 80%, we estimated a required sample per group of about 1300 participants. Considering a 1:1 allocation, the total sample required for this study was approximately 2600 participants (2×1300=2600).

### Data Analysis

#### Data Cleaning

Using data-cleaning techniques to identify careless responses is recommended for internet-based surveys as inattentive responses represent a threat to data validity [58]. We used 2 methods to identify careless responses using the database received from Dynata. First, amongst both the video and text groups, we excluded participants who spent less than 273 seconds or more than 2401 seconds on the survey (lowest and highest 5% of time spent on the survey compared to the mean, 699 seconds). Next, we used responses to the TEQ to identify straight-liners (ie, exhibited no variance in their responses across scale items) and excluded them from subsequent analyses. We chose this scale because it included reverse-coded items, thereby making it highly unlikely that a participant would provide the same response for all items.

#### Statistical Analyses

For baseline sociodemographics, we calculated proportions and means (and SD) and used the Pearson chi-square test and the Welch 2-sample *t* test to evaluate whether the 2 study groups differed significantly. At baseline and postintervention and for each of the study groups, we calculated the proportion of participants in each of the 4 PAPM intention stages (ie, *unengaged*, *undecided*, *decided not*, and *decided to*). For each study group, we calculated the pre- to posttransitions in intentions to receive the COVID-19 vaccine. To estimate the pre- to postintervention change in vaccine intentions, we used a binary outcome (ie, “intenders” corresponding to the stage *decided to* and “nonintenders” that included stages *unengaged*, *undecided*, and *decided not*) and the McNemar chi-square test. To estimate pre-post changes in PAPM intention stages, we conducted exact tests of symmetry (4×4 contingency tables) that comprise pairwise McNemar tests (using the *nominalSymmetryTest* function available in the R package *rcompanion*) [59]. We reported adjusted *P* values for multiple comparisons (Benjamini and Hochberg method), odds ratios (ORs), and the Cohen g effect size that was interpreted as small (0.05 to <0.15), medium (0.15 to <0.25), or large (≥0.25). For each study group, we used the significant transitions between vaccine intention stage pairs for calculating the total number of participants who changed toward increased vaccination intentions (eg, from *undecided* to *decided to*) and estimated the between-group difference using the chi-square 2-sample test for equality of proportions. To estimate the between-group difference in vaccine intentions, the Pearson chi-square Test was conducted on postintervention vaccine intentions using the 4-stage PAPM outcome.

#### Additional Analyses

Using the same analysis approach, we performed 2 subgroup analyses that included (1) all participants who answered the postintervention COVID-19 vaccine intentions question and participants who were initially removed during data cleaning (N=1654) and (2) all participants who were randomly allocated to the study groups and who answered the preintervention COVID-19 vaccine intentions question (N=2089, intention-to-treat approach). In addition, for both subgroups, we performed exploratory between-group analyses and operationalized the vaccine intention outcome in 2 different ways: (1) baseline (preintervention) vaccine intentions in the text group and postintervention intentions in the video group and (2) postintervention vaccine intentions in the text group and baseline intentions in the video group.

All statistical analyses were conducted using R v. 4.0.5 (R Core Team) [60].

### Ethical Considerations

The study was approved by the Research Ethics Board of the Integrated Health and Social Services University Network for West-Central Montreal (CIUSSS West-Central Montreal; Project ID #2021-2732).

## Results

### Participant Flow

**Figure 1:**
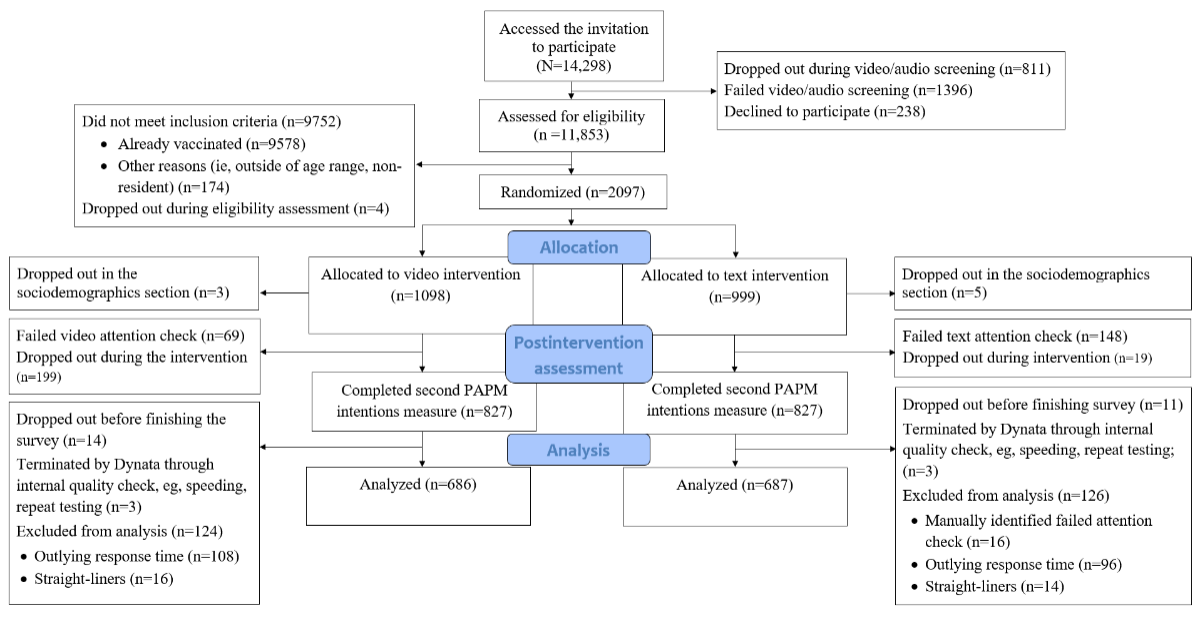
CONSORT diagram. CONSORT: Consolidated Standards of Reporting Trials; PAPM: Precaution Adoption Process Model.

Of 14,298 participants in the target age group who accessed the invitation to participate, 11,853 (82.9%) were assessed for eligibility, of whom 2097 (17.7%) were eligible (n=9578, 80.8%, were excluded because they were already vaccinated against COVID-19; n=174, 1.5%, did not meet other inclusion criteria; and n=4, 0.03%, dropped out) and were randomly allocated to the study arms: 1654 (78.9%) completed the postintervention assessment, and 1373 (65.5%; ie, 686, 50%, and 687, 50%, in the video and text intervention arms, respectively) were included in the analyses. See Figure 1.

### Recruitment Dates and Reasons for Stopping the Trial

Data collection took place from July 30 to September 13, 2021. At about 5 weeks into data collection, daily recruitment significantly declined. The main barrier was the relative low proportion (about 22%) of eligible participants (ie, unvaccinated in the age group of 20-39 years). We conducted preliminary analyses using a total sample of 1346 participants (673, 50%, per group) and found that the number of observations ensured 80% power to detect a 5% pre-post change in vaccine intentions. Preliminary analyses showed a difference of about 2% as opposed to the expected between-group difference of 5% in vaccine intentions that we had anticipated. To reach a similar level of power would have required about 5500 participants per group (ie, an increase of 4200 from our initial sample calculations) to detect a statistically significant superiority of the video intervention. Reaching the new sample size target would not have been feasible due to time and budget considerations, and we decided to stop data collection.

### Baseline Data

The sample consisted of slightly more females (n=740, 53.9%), the mean age was 30.7 years, the majority used English as the primary language at home (n=1122, 81.7%), most reported a total gross household income in the year preceding the pandemic of less than CA $75,000 (US $58563.80, n=848, 61.8%), and most resided in an urban area (n=1067, 77.7%). None of the sociodemographic characteristics differed significantly between the study groups (see Table 1 and Multimedia Appendix 3 for additional subgroup analyses). In the video group, 86 (12.5%) intended to receive the vaccine, 292 (42.6%) were decided against vaccination, 234 (34.1%) were undecided, and 74 (10.8%) had not thought about receiving the COVID-19 vaccine (ie, *unengaged*). Participants allocated to the active control group (text intervention) reported similar vaccine intentions, and the difference between groups was not statistically significant: *χ*^2^_3_=1.62, *P*=.65; see Table 2.

**Table 1:** Sociodemographic variables.

Characteristics		Total (N=1373)	Video group (N=686)	Text group (N=687)	Between-group difference^a^ *P* value	
Age, mean (SD)		30.7 (5.3)	30.7 (5.4)	30.7 (5.3)	.94	
**Sex, n (%)**						.98
	Male	633 (46.1)	316 (46.1)	317 (46.1)	—^b^	
	Female	740 (53.9)	370 (53.9)	370 (53.9)	—	
**Gender, n (%)**						.98
	Man	626 (45.6)	311 (45.3)	315 (45.9)	—	
	Woman	721 (52.5)	362 (52.8)	359 (52.3)	—	
	Gender diverse	26 (1.9)	13 (1.9)	13 (0.4)	—	
**Canadian region, n (%)**						.08
	Western	451 (32.8)	225 (32.8)	226 (32.9)	—	
	East	105 (7.7)	40 (5.8)	65 (9.5)	—	
	Central	813 (59.2)	419 (61.1)	394 (57.3)	—	
	Territories	4 (0.3)	2 (0.3)	2 (0.3)	—	
**Place of residence, n (%)**						.43
	Rural	306 (22.3)	159 (23.2)	147 (21.4)	—	
	Urban	1067 (77.7)	527 (76.8)	540 (78.6)	—	
**Self-perceived visible minority, n (%)**						.05
	Yes	401 (29.2)	217 (31.6)	184 (26.8)	—	
	No	972 (70.8)	469 (68.4)	503 (73.2)	—	
**Language spoken at home, n (%)**						.46
	English	1122 (81.7)	561 (81.8)	561 (81.7)	—	
	French	203 (14.8)	105 (15.3)	98 (14.2)	—	
	Other	48 (3.5)	20 (2.9)	28 (4.1)	—	
**Education (any postsecondary), n (%)**						.63
	Yes	858 (62.5)	433 (63.1)	425 (61.9)	—	
	No	515 (37.5)	253 (36.9)	262 (38.1)	—	
**Income (CA $)^c^, n (%)**						.56
	<19,999 (US $15,616.20)^d^	149 (10.9)	72 (10.5)	77 (11.2)	—	
	20,000-39,999 (US $15,617-$31,233.20)	253 (18.4)	136 (19.8)	117 (17.0)	—	
	40,000-59,999 (US $31,224-$46,850.20)	227 (16.5)	113 (16.5)	114 (16.6)	—	
	60,000-79,999 (US $46,851-$62,467.20)	217 (15.8)	109 (15.9)	108 (15.7)	—	
	80,000-99,999 (US $62,468-$78,084.20)	188 (13.7)	82 (12.0)	106 (15.5)	—	
	>100,000 (US $78,085)	288 (21.0)	148 (21.5)	140 (20.4)	—	
	Prefer not to answer	51 (3.7)	26 (3.8)	25 (3.6)	—	
**Ethnicity, n (%)**						.31
	North American Aboriginal	107 (7.8)	62 (9.0)	45 (6.6)	—	
	Other North American	637 (46.4)	303 (44.2)	334 (48.6)	—	
	European	320 (23.3)	160 (23.3)	160 (23.3)	—	
	Asian	98 (7.1)	51 (7.4)	47 (6.8)	—	
	Other	211 (15.4)	110 (16.0)	101 (14.7)	—	
**Identification as a parent, n (%)**						.89
	Yes	697 (50.8)	347 (50.6)	350 (50.9)	—	
	No	676 (49.2)	339 (49.4)	337 (49.1)	—	

^a^Chi-square or *t* test.

^b^—: not applicable.

^c^Of 1373 participants, 848 (61.8%) and 525 (38.2%) reported an annual income before taxes of all members of the household before the pandemic of <CA $75,000 and ≥CA $75,000, respectively. The between-group difference in proportions was not significant (*P*=.48).

^d^An exchange rate of CA $1=US $0.78 has been applied.

**Table 2:** Number of participants by PAPM^a^ vaccine intention stage and intervention group at baseline and postintervention (N=1373).

Group		Unengaged	Undecided	Decided not	Decided to	Total	Between-group difference^b^ *P* value	
**Baseline, n (%)**								.65
	Video	74 (10.8)	234 (34.1)	292 (42.6)	86 (12.5)	686 (50.0)	—^c^	
	Text	73 (10.6)	255 (37.1)	272 (39.6)	87 (12.7)	687 (50.0)	—	
**Postintervention,** **n (%)**								.64
	Video	54 (7.9)	236 (34.4)	277 (40.4)	119 (17.3)	686 (50.0)	—	
	Text	47 (6.8)	249 (36.2)	285 (41.5)	106 (15.4)	687 (50.0)	—	

^a^PAPM: Precaution Adoption Process Model.

^b^Chi-square test.

^c^—: not applicable.

### Outcomes

In the video group, 43 (6.3%) participants changed from nonintenders at baseline (ie, *unengaged*, *undecided*, or *decided not*) to vaccine intenders (ie, *decided to*) postintervention and 10 (1.5%) participants changed from vaccine intenders at baseline to nonintenders postintervention. The McNemar test was significant (*χ*^2^_1_=20.55, *P*<.001). In the active control (text) group, 24 (3.5%) participants changed from nonintenders at baseline to vaccine intenders postintervention and 5 (0.7%) participants changed from vaccine intenders at baseline to nonintenders postintervention. Unexpectedly, the McNemar test was also significant (*χ*^2^_1_=12.45, *P*<.001).

**Figure 2:**
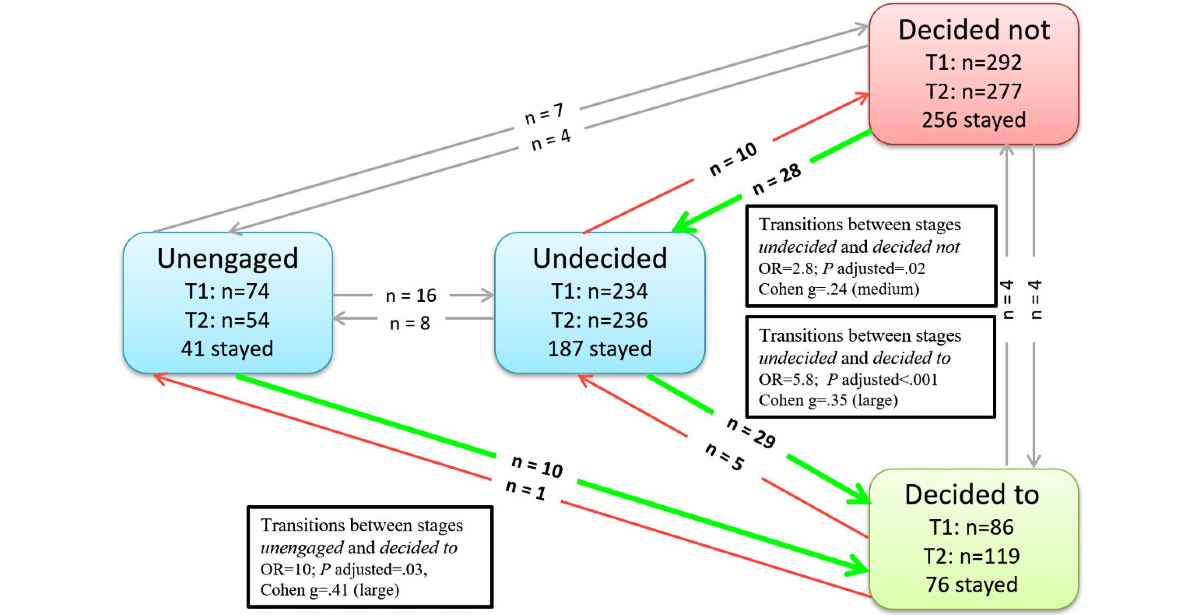
PAPM stage transitions from T1 (baseline) to T2 (postintervention) in the video group (N=686). OR: odds ratio; PAPM: Precaution Adoption Process Model. Green arrows show significant transitions toward increased and red arrows toward decreased vaccination intentions. Gray arrows show nonsignificant transitions between stages..

In the video group, we found a statistically significant change from *decided not* at baseline to *undecided* postintervention (n=28, 4.1%; *P*=.02, OR 2.8, Cohen g=.24), from *undecided* to *decided to* (n=29, 4.2%; *P*<.001, OR 5.8, Cohen g=.35), and from *unengaged* to *decided to* (n=10, 1.5%; *P*=.03, OR 10, Cohen g=.41). In total, in the video group, 67 significant changes toward increased vaccination intentions were observed (see Figure 2 for a visual representation of PAPM stage transitions from baseline to postintervention in the video group and Tables S1 and S2 in Multimedia Appendix 4).

**Figure 3:**
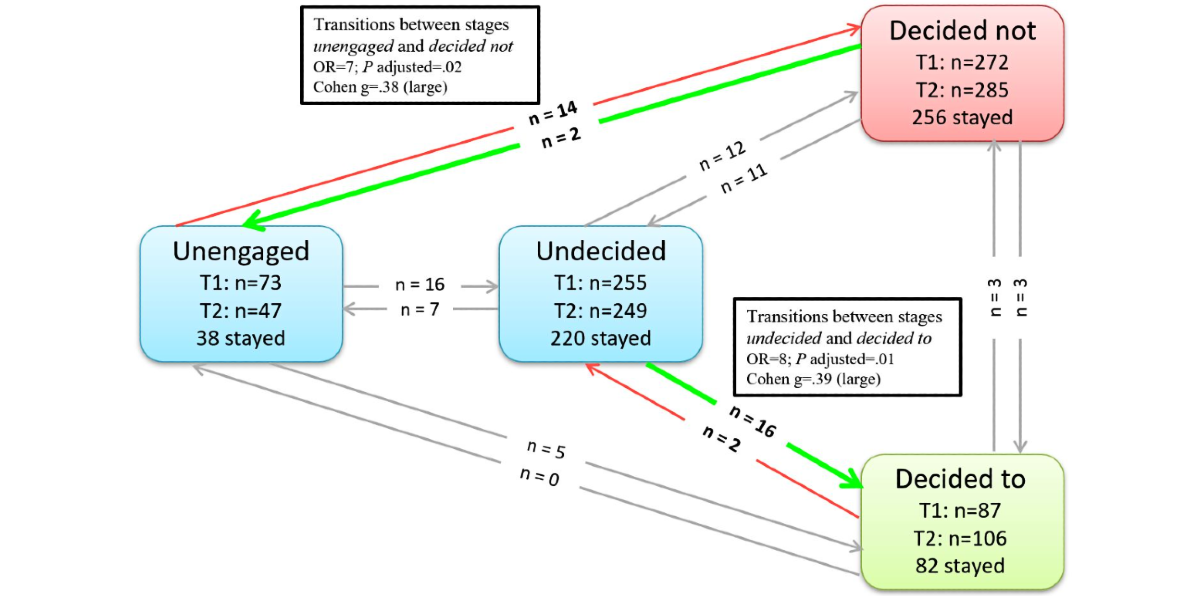
PAPM stage transitions from T1 (baseline) to T2 (postintervention) in the text group (N=687). OR: odds ratio; PAPM: Precaution Adoption Process Model. Green arrows show significant transitions toward increased and red arrows toward decreased vaccination intentions. Gray arrows show nonsignificant transitions between stages.

In the text group, we found a statistically significant change from *unengaged* at baseline to *decided not* postintervention (denoting a change toward decreased vaccine intentions; n=14, 2%; *P*=.02, OR 7, Cohen g=.38) and from *undecided* to *decided to* (n=16, 2.3%; *P*=.01, OR 8, Cohen g=.39). In other words, in the text group, 14 (2%) participants moved toward decreased intentions and 16 (2.3%) participants moved toward increased vaccination intentions (see Figure 3 for a visual representation of PAPM stage transitions from baseline to postintervention in the text group and Tables S1 and S3 in Multimedia Appendix 4). We found a significant difference between those who changed toward increased vaccine intentions in the video group (n=67, 9.77%) compared to the text group (n=16, 2.33%): *χ*^2^_1_=33.43, *P*<.001.

Postintervention, in the video group, 119 (17.3%) intended to receive the vaccine, 277 (40.4%) were decided against vaccination, 236 (34.4%) were undecided, and 54 (7.9%) reported being unengaged. In the text group, 106 (15.4%) intended to receive the vaccine, 285 (41.5%) were decided against vaccination, 249 (36.2%) were undecided, and 47 (6.8%) reported being unengaged. The between-group difference in vaccine intentions was not significant: *χ*^2^_3_=1.70, *P*=.64.

Results of additional subgroup analyses did not significantly differ from per protocol analyses (see Multimedia Appendix 5). The only difference consisted in the loss of statistical significance of the transition from *unengaged* to *decided to* in the video group (see Table S2 in Multimedia Appendix 5) that could be explained by 2 additional participants who transitioned from *decided to* at baseline to *unengaged* postintervention. Since one cannot change from *decided to* get the vaccine to *unengaged*, this was an artifact introduced by careless responding.

Results of exploratory analyses provided a signal that the video intervention was superior to the text intervention as the between-group difference in vaccine intentions was significant when using preintervention intentions in the text group and postintervention intentions in the video group (*χ*^2^_1_=5.90, *P*=.02) and not significant when using preintervention intentions in the video group and postintervention intentions in the text group (*χ*^2^_1_=2.39, *P*=.12); see Tables S10 and S11 in Multimedia Appendix 5. The same results were obtained using samples comprising 1654 (all completers of the second vaccination intention assessment; see Tables S5 and S6 in Multimedia Appendix 5) and 2089 (intention-to-treat) participants (see Tables S8 and S9 in Multimedia Appendix 5).

## Discussion

### Principal Findings

To the best of our knowledge, this is the first population-based study that examined the effect of a video-based intervention eliciting prosocial (altruistic) motives on intentions to receive the COVID-19 vaccine in younger Canadian adults. We used a pre-post and randomized control trial (RCT) study design and recruited a national sample of unvaccinated 20-39-year-old Canadians who participated in a web-based survey between July and September 2021 in the context of the fourth COVID-19 pandemic wave. Our study had 2 specific objectives: (1) to estimate pre- to postintervention vaccine intention changes in participants who were randomly allocated to the video intervention or the text-based intervention that provided non-vaccine-related preventive health measures and (2) to estimate between-group vaccine intentions postintervention.

### Comparison With Prior Work

First, we found that the video intervention was effective in changing vaccine intentions and that 4.8% more participants intended to receive the vaccine postintervention. The size of the effect is consistent with results of the experimental study conducted by Li et al [28], who studied 3952 participants (median age range 31-40 years) from 8 countries (China, France, Japan, United Kingdom, United States, Israel, Brazil, and South Africa) who participated in an internet survey in 2013 before the start of the flu season at the time. They reported a 6% absolute increase in intentions to receive the influenza vaccine in participants who were exposed to prosocial (altruism) messages [28]. Understanding the evolving context in which our study was conducted could explain the modest (4.8%) increase in vaccine intentions. At the time of data collection (July 30-September 13, 2021), about 3 months had elapsed since adults 20-39 years old became eligible to receive the COVID-19 vaccine in Canada. Three-quarters of them had received at least 1 dose [34]. In surveys conducted before the start of vaccination, approximately 40% of our target population was vaccine hesitant compared to 87% who reported vaccine hesitancy in our analyzed sample who are more resistant to vaccination. Therefore, it is possible that had this study been conducted 2 months earlier, our results would have shown a higher increase in pre- to postintervention vaccine intentions. Surprisingly, vaccine intentions also significantly increased in the group that received information about nonvaccine preventive measures in text format, although the effect was smaller than that in the video group, as only 2.7% reported higher vaccine intentions postintervention. Because we used a vaccine-neutral intervention in the active control group, it is possible that the increase represents social desirability. Since we did not measure social desirability, it is possible this bias was also present in the video intervention group as the video depicted vaccination as a social benefit.

Using the theoretical PAPM to inform the measurement of vaccine intentions, we found a more nuanced understanding of pre- to postintervention change in vaccine intentions. Our results show that significantly more participants who watched the video changed toward a more advanced vaccine decision stage than participants in the text group. In both groups, we found that individuals who had not thought about receiving the vaccine (*unengaged*) and those who were *undecided* were more likely to change their intentions to *decided to* vaccinate compared to those who reported being *decided not* at baseline, and this effect was more pronounced in the video group. This pattern of decision-making changes aligns with our previous findings from a longitudinal study evaluating human papillomavirus (HPV) vaccine intention change over a 9-month period in parents of 9-16-year-old boys and girls [22]. In that study, we demonstrated that parents who were *unengaged* or *undecided* at baseline were more likely to increase their HPV vaccine acceptability over time and deemed “flexible hesitant” (ie, changed to *decided to* vaccinate or vaccinated their child). This was in contradistinction with parents who were initially in the *decided not* stage and remained *decided not* over time, whom we deemed as “rigid hesitant” [22]. Therefore, investigating vaccine hesitancy as a binary outcome does not convey the nuances of movement in vaccine intention stages. For individuals who are “flexible hesitant,” viewing messages that highlight altruism may provide the necessary “push” to move toward adoption stages of accepting the vaccine. This could reflect behavioral nudging, in which promoting the positive impacts of a behavior without changing incentives or forbidding negative options can have a substantial impact on the behavior [61,62]. A recent systematic review by Reñosa et al [63] found that nudging messages that invoked emotional affect, such as storytelling and dramatic narratives, can improve vaccine confidence and uptake. In addition, Wood and Schulman (2021) [64] suggested that apathy toward vaccination, a characteristic that might contribute to someone being *unengaged*, could be addressed with peripheral, emotional messaging to motivate behavior change. Interestingly, in the video group, significantly more people moved from *decided not* to *undecided*, suggesting that the evocation of concern for others (altruism) may prompt even “rigid” hesitant individuals to reflect and rethink their decision.

Although pre-post analyses showed that the video intervention was effective in increasing vaccine intentions, between-group analyses did not confirm our hypothesis that watching the video would result in statistically significant higher intentions compared to reading non-vaccine-related information. Two factors may have contributed to this outcome: (1) The unexpected 2.7% increase in vaccine intentions in the active control group that reduced the hypothesized 5% between-group difference, and (2) the higher-than-expected vaccine hesitancy in our sample (which comprised ~40% “rigid hesitant” compared to ~10% found in 2 population-based studies conducted by our team that investigated HPV vaccine hesitancy [21,65]) that could have attenuated the effect of the video on vaccine intentions because “rigid hesitant” are less amenable to changes in intentions.

Although achieving statistical significance for the between-group difference would have sent a strong signal related to the efficacy of the video intervention, we believe that our study can inform future research using interventions that elicit prosocial motives to increase COVID-19 vaccine intentions. For example, interventions could be adapted to include other forms of prosocial motivations, such as collectivism (the practice of prioritizing a group over individuals within the group) [66]. Previous research has shown that collectivism is associated with COVID-19 vaccine acceptance [12,67], while individualism (ie, emphasis on the autonomous individual) is associated with COVID-19 vaccine hesitancy [68]. Therefore, to override feelings of personal invulnerability to COVID-19 in countries that are more individualistic than collectivistic (eg, Canada, the United States), messages that promote community well-being, highlight shared goals, and induce feelings of interdependence should be used to encourage COVID-19 vaccination [69]. Importantly, the design of our intervention aligns well with the recommendations for animated, video-based health communication interventions published by Adam et al [70] in 2021. Our intervention used a narrative approach, was well adapted to the Canadian cultural context as it was available in English and French, used characters of different ages and ethnic backgrounds, used appealing colors that ensured an optimal contrast independent of the size of the screen, included the voice of a narrator with experience in media communications, and had a length aligned with the recommend optimal length of around 2.5 minutes [70].

### Limitations

The main limitations derive from the premature termination of the study dictated by barriers in participant recruitment and by lower-than-anticipated COVID-19 vaccine hesitancy in the population of interest. As the target sample size was not reached, the sampling quotas used to match Canadian Census data deviated from the planned quotas and we included 3.9% more females, 2.3% more participants residing in rural areas, 5.2% less Francophones, and 11.8% less participants with annual total income before taxes of all members of the household before the pandemic of CA $75,000 (US $58,563.80). Although between-group differences were not significant, these differences in sociodemographics could impede the generalizability of the results to the Canadian population. The high proportion of participants who were in the *decided not* to vaccinate stage could have diminished our ability to prove the superiority of the video intervention in increasing vaccine intentions. Additionally, the use of an active control group could have diminished our capacity to prove the statistical superiority of the video intervention, perhaps due to social desirability. Finally, follow-up 3-6 months later would have allowed us to evaluate the translation of increased vaccine intentions into actual vaccine uptake.

### Conclusion

Using a web-survey and a pre-post and RCT study design, we showed that a brief video eliciting prosocial (altruism) motives increased COVID-19 vaccine intentions of Canadians aged 20-39 years, especially among those who were less engaged in the decision to vaccinate or were undecided. As web streaming is highly popular among younger adults, using short videos is an efficient modality to disseminate public health messages. The effect of the new intervention on increasing intentions was modest, but delivering messages that elicit prosocial motives to vaccinate to a large population could increase vaccine intentions in a significant number of individuals and assist in reaching vaccination targets and curbing the effect of the pandemic. As vaccine hesitancy is complex, it is likely that a multifaceted messaging approach that includes the benefits of vaccination for the community would be beneficial, especially in societies where individual values prevail over collective values. Our intervention could be adapted to align with the latest COVID-19 immunization recommendations (eg, boosters) or to increase vaccine intentions for other preventable diseases.

## Appendix

Multimedia Appendix 1 Strata.

Multimedia Appendix 2 Text intervention.

Multimedia Appendix 3 Sociodemographics subgroups.

Multimedia Appendix 4 Results tables.

Multimedia Appendix 5 Additional analyses.

Multimedia Appendix 6 CONSORT-eHEALTH checklist (V 1.6.1).

## Abbreviations

CONSORT: Consolidated Standards of Reporting Trials
HPV: human papillomavirus
K_6_: 6-item Kessler Psychological Distress Scale
OR: odds ratio
PTM: prosocial tendencies measure
PAPM: Precaution Adoption Process Model
RCT: randomized control trial
TEQ: Toronto Empathy Questionnaire
