# Supplementary figures and images for: The Efficacy of a Brief, Altruism-Eliciting Video Intervention in Enhancing COVID-19 Vaccination Intentions Among a Population-Based Sample of Younger Adults: Randomized Controlled Trial
Source: JMIR Public Health Surveill. 2022 May 30;8(5):e37328. doi: 10.2196/37328 (PMC9153910; doi:10.2196/37328)

Multimedia Appendix 1. Strata.


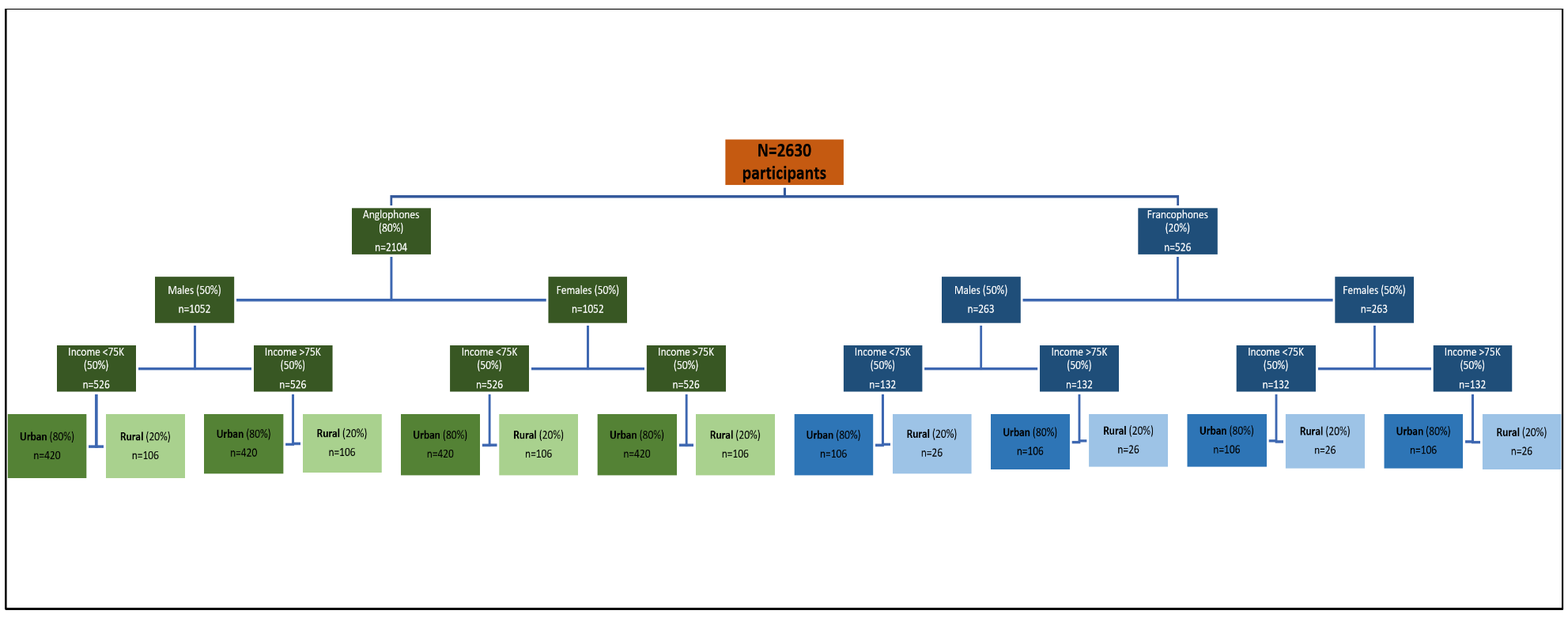

Supplement: Multimedia Appendix 1 [file publichealth_v8i5e37328_app1.docx]
