# Supplementary material for: The Efficacy of a Brief, Altruism-Eliciting Video Intervention in Enhancing COVID-19 Vaccination Intentions Among a Population-Based Sample of Younger Adults: Randomized Controlled Trial
Source: JMIR Public Health Surveill. 2022 May 30;8(5):e37328. doi: 10.2196/37328 (PMC9153910; doi:10.2196/37328)
Supplement: Multimedia Appendix 2 [file publichealth_v8i5e37328_app2.docx]

**Multimedia Appendix 2. Text intervention.**

**Text intervention and attention check questions**

COVID-19 spreads from an infected person to others through respiratory droplets and aerosols (smaller droplets) created when an infected person talks, sings, shouts, coughs, or sneezes. COVID-19 can also spread by touching something that has the virus on it, then touching your mouth, nose or eyes with unwashed hands. You can transmit COVID-19 even before you start showing symptoms or without ever developing symptoms.

The Public Health Agency of Canada recommends practicing several preventive health behaviours to reduce the spread of COVID-19 disease. Here is a reminder of what these recommended behaviours are:

**General hygiene**

In your day-to-day activities, you can reduce the risk of infection and or spreading infection to others by doing the following:

- Wash your hands often, with soap and warm water for at least 20 seconds
- Cough or sneeze into a tissue or the bend of your arm (not your hand)
- Avoid touching your eyes, nose, or mouth with unwashed hands
- Wear a mask when in a shared space (both indoors and outdoors)
- Regularly clean high-touch surfaces such as door handles, phones, television remotes, and toilets with regular household cleaners or diluted bleach
- Ensure proper ventilation to reduce potentially infectious particles in the air indoors.

*Quiz/check:*

*Please select the statement that was* ***not*** *explained in the text you have just read:*

1. *Cleaning high touch surfaces*
2. *Washing your hands often, with soap and warm water*
3. *Wearing a mask when in a shared space*
4. *Limit sexual activity with new partners*

**Physical distancing**

Public health recommends keeping your contact with people outside your household to a minimum. This includes:

- Avoiding closed spaces with poor ventilation and crowded places
- Staying home and away from others if you feel sick
- Keeping the number of people you have prolonged contact with as small as possible
- Sticking to a small and consistent social circle and avoiding gathering in large groups
- Talking to your employer about working at home if possible
- Limiting contact with those at risk of more severe illness such as older adults, those with underlying medical conditions, and those with compromised immune systems
- Maintaining a physical distance of 2 meters from people outside your household
- Limiting sexual activity with new partners.

*Quiz/check:*

*Please select the statement that was* ***not*** *explained in the text you have just read:*

1. *Staying home and away from others if you feel sick*
2. *Avoiding gathering in large groups*
3. *Providing proof of a negative COVID-19 test before returning to Canada after travelling*
4. *Limit contact with those at risk of more severe illness*

**Travel/quarantine/isolation**

Public health recommends avoiding all non-essential travel outside of Canada as travelling increases your risk of getting infected with COVID-19. If you are planning to return to Canada, you may be required to take a COVID-19 test and provide proof of a negative COVID-19 test result before entering Canada.

Upon your return to Canada (as well as for return from inter-provincial travel between certain provinces/territories), you may be required to:

- Quarantine (self-isolate) at home when you may have been exposed to COVID-19 and have no symptoms
- Isolate at home if you have been diagnosed with COVID-19, you have symptoms of COVID-19, or if you are waiting to hear results of a COVID-19 lab test

*Quiz/check:*

*Please select the statement that was* ***not*** *explained in the text you have just read:*

1. *Avoiding all non-essential travel*
2. *Engaging in physical activity outdoors instead of indoors*
3. *Taking a COVID-19 test before entering Canada*
4. *Isolating at home if you have symptoms of COVID-19 upon your return to Canada*
