# Supplementary material for: The Efficacy of a Brief, Altruism-Eliciting Video Intervention in Enhancing COVID-19 Vaccination Intentions Among a Population-Based Sample of Younger Adults: Randomized Controlled Trial
Source: JMIR Public Health Surveill. 2022 May 30;8(5):e37328. doi: 10.2196/37328 (PMC9153910; doi:10.2196/37328)
Supplement: Multimedia Appendix 3 [file publichealth_v8i5e37328_app3.docx]

**Multimedia Appendix 3. Sociodemographics subgroups.**

**Table 1.** Sociodemographics, all participants who responded to the baseline vaccine intentions question (N=2089)

|  | | **Total**  **(N = 2089)** | **Video group**  **(n = 1095)** | **Text group**  **(n = 994)** | **Between group difference*** |
| --- | --- | --- | --- | --- | --- |
|  |  | n (%) or Mean (SD) | n (%) or Mean (SD) | n (%) or Mean (SD) |  |
| **Age** | | 30.6 (5.2) | 30.8 (5.2) | 30.4 (5.2) | *P*=.11 |
| **Sex** | |  |  |  |  |
|  | Male | 1019 (48.8) | 530 (48.4) | 489 (49.2) | *P*=.72 |
|  | Female | 1070 (51.2) | 565 (51.6) | 505 (50.8) |  |
| **Gender** | |  |  |  |  |
|  | Man | 1025 (49.1) | 532 (48.6) | 493 (49.6) | *P*=.86 |
|  | Woman | 1026 (49.1) | 542 (49.5) | 484 (48.7) |  |
|  | Gender diverse | 38 (1.8) | 21 (1.9) | 17 (1.7) |  |
| **Canadian region** | |  |  |  |  |
|  | Western | 702 (33.6) | 373 (34.1) | 329 (33.1) | *P*=.04 |
|  | Central | 1207 (57.8) | 640 (58.4) | 567 (57.0) |  |
|  | Eastern | 163 (7.8) | 70 (6.4) | 93 (9.4)* |  |
|  | Territories | 17 (0.8) | 12 (1.1) | 5 (0.5) |  |
| **Place of residence** | |  |  |  |  |
|  | Rural | 561 (26.9) | 315 (28.8) | 246 (24.7) | *P*=.04¶ |
|  | Urban | 1528 (73.1) | 780 (71.2) | 748 (75.3) |  |
| **Self-perceived visible minority** | |  |  |  |  |
|  | Yes | 739 (35.4) | 398 (36.3) | 341 (34.3) | *P*=.33 |
|  | No | 1350 (64.6) | 697 (63.7) | 653 (65.7) |  |
| **Language spoken at home** | |  |  |  |  |
|  | English | 1761 (84.3) | 929 (84.8) | 832 (83.7) | *P*=.50 |
|  | French | 262 (12.5) | 136 (12.4) | 126 (12.7) |  |
|  | Other | 66 (3.2) | 30 (2.7) | 36 (3.6) |  |
| **Education (any post-secondary)** | |  |  |  |  |
|  | Yes | 1381 (66.1) | 718 (65.6) | 663 (66.7) | *P*=.59 |
|  | No | 708 (33.9) | 377 (34.4) | 331 (33.3) |  |
| **Income (CAD)** | |  |  |  |  |
|  | <19999 | 216 (10.3) | 109 (10.0) | 107 (10.8) | *P*=.88 |
|  | 20000-39999 | 362 (17.3) | 196 (17.9) | 166 (16.7) |  |
|  | 40000-59999 | 312 (14.9) | 157 (14.3) | 155 (15.6) |  |
|  | 60000-79999 | 352 (16.9) | 182 (16.6) | 170 (17.1) |  |
|  | 80000-99999 | 318 (15.2) | 164 (15.0) | 154 (15.5) |  |
|  | >100000 | 444 (21.3) | 239 (21.8) | 205 (20.6) |  |
|  | Prefer not to answer | 85 (4.1) | 48 (4.4) | 37 (3.7) |  |
| **Ethnicity** | |  |  |  |  |
|  | North American Aboriginal | 221 (10.6) | 125 (11.4) | 96 (9.7) | *P*=.54 |
|  | Other North American | 969 (46.4) | 491 (44.8) | 478 (48.1) |  |
|  | European | 444 (21.3) | 235 (21.5) | 209 (21.0) |  |
|  | Asian | 148 (7.1) | 78 (7.1) | 70 (7.0) |  |
|  | Other | 307 (14.7) | 166 (15.2) | 141 (14.2) |  |
| **Identification as a parent** | | | | | |
|  | Yes | 1171 (56.1) | 621 (56.7) | 550 (55.3) | *P*=.53 |
|  | No | 918 (43.9) | 474 (43.3) | 444 (46.7) |  |

Obs: * denotes effect size, Cohen h=.11 (very small); ¶ denotes Cohen h=.09 (very small)

**Table 2.** Sociodemographics, all participants who responded to the post-intervention vaccine intentions question, including participants who were flagged as careless responders during data cleaning (N=1654)

|  | | **Total**  **(N = 1654)** | **Video group**  **(n = 827)** | **Text group**  **(n = 827)** | **Between group difference*** |
| --- | --- | --- | --- | --- | --- |
|  |  | n (%) or Mean (SD) | n (%) or Mean (SD) | n (%) or Mean (SD) |  |
| **Age** | | 30.7 (5.3) | 30.8 (5.3) | 30.6 (5.3) | *P*=.41 |
| **Sex** | |  |  |  |  |
|  | Male | 759 (45.9) | 381 (46.1) | 378 (45.7) | *P*=.88 |
|  | Female | 895 (54.1) | 446 (53.9) | 449 (54.3) |  |
| **Gender** | |  |  |  |  |
|  | Man | 752 (45.5) | 376 (45.5) | 376 (45.5) | *P*=.98 |
|  | Woman | 871 (52.7) | 436 (52.7) | 435 (52.6) |  |
|  | Gender diverse | 31 (1.9) | 15 (1.8) | 16 (1.9) |  |
| **Canadian region** | |  |  |  |  |
|  | Western | 523 (31.6) | 265 (32.0) | 258 (31.2) | *P*=.02 |
|  | Central | 997 (60.3) | 511 (61.8) | 486 (58.8) |  |
|  | Eastern | 127 (7.7) | 47 (5.7) | 80 (9.7)* |  |
|  | Territories | 7 (0.4) | 4 (0.5) | 3 (0.4) |  |
| **Place of residence** | |  |  |  |  |
|  | Rural | 383 (23.2) | 191 (23.1) | 192 (23.2) | *P*=.95 |
|  | Urban | 1271 (76.8) | 636 (76.9) | 635 (76.8) |  |
| **Self-perceived visible minority** | |  |  |  |  |
|  | Yes | 493 (29.8) | 253 (30.6) | 240 (29.0) | *P*=.49 |
|  | No | 1161 (70.2) | 574 (69.4) | 587 (71.0) |  |
| **Language spoken at home** | |  |  |  |  |
|  | English | 1367 (82.6) | 685 (82.8) | 682 (82.5) | *P*=.64 |
|  | French | 228 (13.8) | 116 (14.0) | 112 (13.5) |  |
|  | Other | 59 (3.6) | 26 (3.1) | 33 (4.0) |  |
| **Education (any post-secondary)** | |  |  |  |  |
|  | Yes | 1053 (63.7) | 526 (63.6) | 527 (63.7) | *P*=.96 |
|  | No | 601 (36.3) | 301 (36.4) | 300 (36.3) |  |
| **Income (CAD)** | |  |  |  |  |
|  | <19999 | 179 (10.8) | 88 (10.6) | 91 (11.0) | *P*=.48 |
|  | 20000-39999 | 302 (18.3) | 161 (19.5) | 141 (17.0) |  |
|  | 40000-59999 | 266 (16.1) | 128 (15.5) | 138 (16.7) |  |
|  | 60000-79999 | 268 (16.2) | 130 (15.7) | 138 (16.7) |  |
|  | 80000-99999 | 232 (14.0) | 105 (12.7) | 127 (15.4) |  |
|  | >100000 | 344 (20.8) | 183 (22.1) | 161 (19.5) |  |
|  | Prefer not to answer | 63 (3.8) | 32 (3.9) | 31 (3.7) |  |
| **Ethnicity** | |  |  |  |  |
|  | North American Aboriginal | 129 (7.8) | 69 (8.3) | 60 (7.3) | *P*=.43 |
|  | Other North American | 757 (45.8) | 359 (43.4) | 398 (48.1) |  |
|  | European | 383 (23.2) | 199 (24.1) | 184 (22.2) |  |
|  | Asian | 133 (8.0) | 70 (8.5) | 63 (7.6) |  |
|  | Other | 252 (15.2) | 130 (15.7) | 122 (14.8) |  |
| **Identification as a parent** | | | | | |
|  | Yes | 860 (52.0) | 431 (52.1) | 429 (51.9) | *P*=.92 |
|  | No | 794 (48.0) | 396 (47.9) | 398 (48.1) |  |

Obs: * denotes effect size, Cohen h=.15 (very small)

**Table 3.** Sociodemographics, participants who dropped between baseline and post intervention assessment of vaccine intentions (N=435)

|  | | **Total**  **(N = 435)** | **Video group**  **(n = 268)** | **Text group**  **(n = 167)** | **Between group difference*** |
| --- | --- | --- | --- | --- | --- |
|  |  | n (%) or Mean (SD) | n (%) or Mean (SD) | n (%) or Mean (SD) |  |
| **Age** | | 30.4 (4.9) | 30.8 (4.8) | 29.8 (5.0) | *P*=.03* |
| **Sex** | |  |  |  |  |
|  | Male | 260 (59.8) | 149 (55.6) | 111 (66.5) | *P*=.02^¶^ |
|  | Female | 175 (40.2) | 119 (44.4) | 56 (33.5) |  |
| **Gender** | |  |  |  |  |
|  | Man | 273 (62.8) | 156 (58.2) | 117 (70.1)♦ | *P*=.03 |
|  | Woman | 155 (35.6) | 106 (39.6) | 49 (29.3)● |  |
|  | Gender diverse | 7 (1.6) | 6 (2.2) | 1 (0.6) |  |
| **Canadian region** | |  |  |  |  |
|  | Western | 179 (41.1) | 108 (40.3) | 71 (42.5) | *P*=.65 |
|  | Central | 210 (48.3) | 129 (48.1) | 81 (48.5) |  |
|  | Eastern | 36 (8.3) | 23 (8.6) | 13 (7.8) |  |
|  | Territories | 10 (2.3) | 8 (3.0) | 2 (1.2) |  |
| **Place of residence** | |  |  |  |  |
|  | Rural | 178 (40.9) | 124 (46.3) | 54 (32.3) | *P*=.004^§^ |
|  | Urban | 257 (59.1) | 144 (53.7) | 113 (67.7) |  |
| **Self-perceived visible minority** | |  |  |  |  |
|  | Yes | 246 (56.6) | 145 (54.1) | 101 (60.5) | *P*=.19 |
|  | No | 189 (43.4) | 123 (45.9) | 66 (39.5) |  |
| **Language spoken at home** | |  |  |  |  |
|  | English | 394 (90.6) | 244 (91.0) | 150 (89.8) | *P*=.91 |
|  | French | 34 (7.8) | 20 (7.5) | 14 (8.4) |  |
|  | Other | 7 (1.6) | 4 (1.5) | 3 (1.8) |  |
| **Education (any post-secondary)** | |  |  |  |  |
|  | Yes | 328 (75.4) | 192 (71.6) | 136 (81.4) | *P*=.02^¥^ |
|  | No | 107 (24.6) | 76 (28.4) | 31 (18.6) |  |
| **Income (CAD)** | |  |  |  |  |
|  | <19999 | 37 (8.5) | 21 (7.8) | 16 (9.6) | *P*=.55 |
|  | 20000-39999 | 60 (13.8) | 35 (13.1) | 25 (15.0) |  |
|  | 40000-59999 | 46 (10.6) | 29 (10.8) | 17 (10.2) |  |
|  | 60000-79999 | 84 (19.3) | 52 (19.4) | 32 (19.2) |  |
|  | 80000-99999 | 86 (19.8) | 59 (22.0) | 27 (16.2) |  |
|  | >100000 | 100 (23.0) | 56 (20.9) | 44 (26.3) |  |
|  | Prefer not to answer | 22 (5.1) | 16 (6.0) | 6 (3.6) |  |
| **Ethnicity** | |  |  |  |  |
|  | North American Aboriginal | 92 (21.1) | 56 (20.9) | 36 (21.6) | *P*=.91 |
|  | Other North American | 212 (48.7) | 132 (49.3) | 80 (47.9) |  |
|  | European | 61 (14.0) | 36 (13.4) | 25 (15.0) |  |
|  | Asian | 15 (3.4) | 8 (3.0) | 7 (4.2) |  |
|  | Other | 55 (12.6) | 36 (13.4) | 19 (11.4) |  |
| **Identification as a parent** | | | | | |
|  | Yes | 311 (71.5) | 190 (70.9) | 121 (72.5) | *P*=.73 |
|  | No | 124 (28.5) | 78 (29.1) | 46 (27.5) |  |

Note: *Cohen d=.22; ¶ Cohen h=.22; ♦ Cohen h=.25; ● Cohen h=.22; § Cohen h=.29; ¥Cohen h=.23

Effect sizes in the range 0.2-0.3 are interpreted as being small.
