# Supplementary material for: The Efficacy of a Brief, Altruism-Eliciting Video Intervention in Enhancing COVID-19 Vaccination Intentions Among a Population-Based Sample of Younger Adults: Randomized Controlled Trial
Source: JMIR Public Health Surveill. 2022 May 30;8(5):e37328. doi: 10.2196/37328 (PMC9153910; doi:10.2196/37328)
Supplement: Multimedia Appendix 4 [file publichealth_v8i5e37328_app4.docx]

**Multimedia Appendix 4. Results tables.**

**Table S1.** Participant PAPM vaccine intention stage transitions from baseline to post intervention for the video and text groups

| **Video group** (n=686) | | | | | |
| --- | --- | --- | --- | --- | --- |
|  | | **Post intervention PAPM stage** | | | |
|  |  | Unengaged | Undecided | Decided not | Decided to |
| **Baseline PAPM stage** | Unengaged (n=74) | 41 | 16 | 7 | **10^*^** |
|  | Undecided (n=234) | 8 | 187 | 10^§^ | **29^¶^** |
|  | Decided not (n=292) | 4 | **28^§^** | 256 | 4 |
|  | Decided to (n=86) | 1^*^ | 5^¶^ | 4 | 76 |
| **Text group** (n=687) | | | | | |
| **Baseline PAPM stage** |  | **Post intervention PAPM stage** | | | |
|  | Unengaged (n=73) | 38 | 16 | **14^*^** | 5 |
|  | Undecided (n=255) | 7 | 220 | 12 | **16^¶^** |
|  | Decided not (n=272) | 2^*^ | 11 | 256 | 3 |
|  | Decided to (n=87) | 0 | 2¶ | 3 | 82 |

Note: Significant transitions between stage pairs are marked with the same symbol. The direction of effect is marked in bold.

**Table S2**. Exact test of symmetry and effect size for the **video group** (n=686)

| McNemar pairwise group symmetry tests  Stage before/after vs. stage before/after | *P* value | *P* adjusted | OR | Probability | Cohen g |
| --- | --- | --- | --- | --- | --- |
| Unengaged vs. undecided | .153 | .229 | 2 | .667 | .167 |
| Unengaged vs. decided not | .546 | .655 | 1.75 | .636 | .136 |
| Unengaged vs. decided to | .0159 | **.0318** | **10** | .909 | **.409** |
| Undecided vs. decided not | .00582 | **.0175** | **2.8** | .737 | **.237** |
| Undecided vs. decided to | <.001 | **<.001** | **5.8** | .853 | **.353** |
| Decided not vs. decided to | 1 | 1 | 1 | .50 | 0 |

Note: In bold significant differences and effect size; OR=odds ratio

**Table S3**. Exact test of symmetry and effect size for the **text group** (n=687)

| McNemar pairwise group symmetry tests  Stage before/after vs. stage before/after | *P* value | *P* adjusted | OR | Probability | Cohen g |
| --- | --- | --- | --- | --- | --- |
| Unengaged vs. undecided | .0953 | .143 | 2.29 | .696 | .196 |
| Unengaged vs. decided not | .00596 | **.0179** | **7** | .875 | **.375** |
| Unengaged vs. decided to | .0736 | .143 | Inf | 1 | .5 |
| Undecided vs. decided not | 1 | 1 | 1.09 | .522 | .0217 |
| Undecided vs. decided to | .00218 | **.0131** | **8** | .889 | **.389** |
| Decided not vs. decided to | 1 | 1 | 1 | .50 | 0 |

Note: In bold significant differences and effect size; OR=odds ratio
