# Supplementary material for: The Efficacy of a Brief, Altruism-Eliciting Video Intervention in Enhancing COVID-19 Vaccination Intentions Among a Population-Based Sample of Younger Adults: Randomized Controlled Trial
Source: JMIR Public Health Surveill. 2022 May 30;8(5):e37328. doi: 10.2196/37328 (PMC9153910; doi:10.2196/37328)
Supplement: Multimedia Appendix 5 [file publichealth_v8i5e37328_app5.docx]

**Multimedia Appendix 5. Additional analyses.**

**Participants who responded to the post-intervention vaccine intentions question, including participants who were flagged as careless responders during data cleaning (N=1654)**

**Table S1.** PAPM vaccine intention stage per group at baseline and post intervention (N=1654)

| **Group** | **Unengaged** | **Undecided** | **Decided not** | **Decided to** | **n (%)** | **Between group difference*** |
| --- | --- | --- | --- | --- | --- | --- |
| **Baseline** n (%) | | | | | | |
| **Video** | 96 (11.6) | 292 (35.3) | 323 (39.1) | 116 (14.0) | 827 (50.0) | *P*=.61 |
| **Text** | 97 (11.7) | 317 (38.3) | 305 (36.9) | 108 (13.1) | 827 (50.0) |  |
| **Post intervention** n (%) | | | | | | |
| **Video** | 77 (9.3) | 295 (35.7) | 304 (36.7) | 151 (18.3) | 827 (50.0) | *P*=.62 |
| **Text** | 77 (9.3) | 296 (35.8) | 322 (38.9) | 132 (16.0) | 827 (50.0) |  |

Note: * denotes Chi-squared test at baseline: X-squared=1.8331, df=3, *P*=.6078, and post intervention: X-squared=1.7949, df=3, *P*=.616

For the binary outcome (Yes/No), pre-intervention: X-squared=.33047, df=1, *P*=.5654; and post intervention: X-squared=1.5389, df=1, *P*=.2148

**Table S2.** PAPM vaccine intention stage transitions from baseline to post intervention for the video and text groups (N=1654)

| **Video group** (n=827) | | | | | |
| --- | --- | --- | --- | --- | --- |
|  | | **Post intervention PAPM stage** | | | |
|  |  | Unengaged | Undecided | Decided not | Decided to |
| **Baseline PAPM stage** | Unengaged (n=96) | 58 | 18 | 9 | 11 |
|  | Undecided (n=292) | 12 | 236 | 10^§^ | **34^¶^** |
|  | Decided not (n=323) | 4 | **33^§^** | 281 | 5 |
|  | Decided to (n=116) | 3 | 8^¶^ | 4 | 101 |
| **Text group** (n=827) | | | | | |
| **Baseline PAPM stage** |  | **Post intervention PAPM stage** | | | |
|  | Unengaged (n=97) | 56 | 18 | **15^*^** | 8 |
|  | Undecided (n=317) | 16 | 261 | 17 | **23^¶^** |
|  | Decided not (n=305) | 2^*^ | 14 | 285 | 4 |
|  | Decided to (n=108) | 3 | 3¶ | 5 | 97 |

Note: Significant transitions between stage pairs are marked with the same symbol. The direction of effect is marked in bold.

Observations:

- In the video group the change pre to post is significant (McNemar's chi-squared=18.846, df=1, *P*<.001)
- In the text group the change pre to post is significant (McNemar's chi-squared=12.522, df=1, *P*<.001)

**Table S3**. Exact test of symmetry and effect size for the **video group** (n=827)

| McNemar pairwise group symmetry tests  Stage before/after vs. stage before/after | *P* value | *P* adjusted | OR | Probability | Cohen g |
| --- | --- | --- | --- | --- | --- |
| Unengaged vs. undecided | .361 | .433 | 1.5 | .6 | .1 |
| Unengaged vs. decided not | .267 | .400 | 2.25 | .692 | .192 |
| Unengaged vs. decided to | .0614 | .123 | 3.67 | .786 | .286 |
| Undecided vs. decided not | <.001 | **.0238** | **3.3 (2.8)** | .767 | **.267 (.237)** |
| Undecided vs. decided to | <.001 | **<.001** | **4.25 (5.8)** | .81 | **.31 (.353)** |
| Decided not vs. decided to | 1 | 1 | 1.25 | .556 | .0556 |

Note: In bold significant differences and effect size; OR=odds ratio

Observation:

- In brackets the OR and effect size on n=686 (corresponding to per protocol analyses, n=1373)

**Table S4**. Exact test of symmetry and effect size for the **text group** (n=827)

| McNemar pairwise group symmetry tests  Stage before/after vs. stage before/after | *P* value | *P* adjusted | OR | Probability | Cohen g |
| --- | --- | --- | --- | --- | --- |
| Unengaged vs. undecided | .864 | 1 | 1.12 | .529 | .0294 |
| Unengaged vs. decided not | .00361 | **.0108** | **7.5 (7)** | .882 | **.382 (.375)** |
| Unengaged vs. decided to | .228 | .456 | 2.67 | .727 | .227 |
| Undecided vs. decided not | .719 | 1 | 1.21 | .548 | .0484 |
| Undecided vs. decided to | <.001 | **.00116** | **7.67 (8)** | .885 | **.385 (.389)** |
| Decided not vs. decided to | 1 | 1 | 1.25 | .556 | .0556 |

Note: In bold significant differences and effect size; OR=odds ratio

- Obs: In brackets the OR and effect size on n=687 (corresponding to n=1373)

**Exploratory between-group analyses (N=1654)**

**Table S5.** Baseline (pre intervention) vaccine intentions in the control group and post intervention intentions in the video group (N=1654)

| **Study group** | **Non-intenders** n(%) | **Intenders** n(%) | **Total** n(%) |
| --- | --- | --- | --- |
| Active control (text) | 719 (86.9) | 108 (13.1 | 827 (50) |
| Video | 676 (81.7) | 151 (18.3) | 827 (50) |
| Total | 1395 | 259 | 1654 |

X-squared=8.4644, df=1, *P*=.003622

**Table S6.** Baseline (pre intervention) vaccine intentions in the video group and post intervention intentions in the control group (N=1654)

| **Study group** | **Non-intenders** n(%) | **Intenders** n(%) | **Total** n(%) |
| --- | --- | --- | --- |
| Active control (text) | 695 (84.0) | 132 (16.0) | 827 (50) |
| Video | 711 (86.0) | 116 (14.0) | 827 (50) |
| Total | 1406 | 248 | 1654 |

X-squared=1.2143, df=1, *P*=.2705

**Participants who responded to the baseline vaccine intentions question (N = 2089, ITT)**

**Table S7.** PAPM vaccine intention stage per group at baseline and post intervention (N=2089)

| **Group** | **Unengaged** | **Undecided** | **Decided not** | **Decided to** | **n (%)** | **Between group difference*** |
| --- | --- | --- | --- | --- | --- | --- |
| **Baseline** n (%) | | | | | | |
| **Video** | 176 (16.1) | 376 (34.3) | 381 (34.8) | 162 (14.8) | 1095 (52.4) | *P*=.48 |
| **Text** | 170 (17.1) | 361 (36.3) | 335 (33.7) | 128 (12.9) | 994 (47.6) |  |
| **Post intervention** n (%) | | | | | | |
| **Video** | 157 (14.3) | 379 (34.6) | 362 (33.1) | 197 (18.0) | 1095 (52.4) | *P*=.34 |
| **Text** | 150 (15.1) | 340 (34.2) | 352 (35.4) | 152 (15.3) | 994 (47.6) |  |

Note: * denotes Chi-squared test at baseline: $\chi^{2}$(3)=2.47, *P*=.480, and post intervention: $\chi^{2}$(3)=3.34, *P*=.342. For the binary intentions outcome preintervention: X-squared=1.602, df=1, *P*=.2056; and postintervention: X-squared=2.7278, df=1, *P*=.09862

**Exploratory between-group analyses (N=2089)**

**Table S8.** Baseline (pre intervention) vaccine intentions in the control group and post intervention intentions in the video group (N=2089)

| **Study group** | **Non-intenders** n(%) | **Intenders** n(%) | **Total** n(%) |
| --- | --- | --- | --- |
| Active control (text) | 866 (87.1) | 128 (12.9) | 994 (47.6) |
| Video | 898 (82.0) | 197 (18.0) | 1095 (52.4) |
| Total | 1764 | 325 | 2089 |

X-squared=10.371, df=1, *P*=.00128

**Table S9.** Baseline (pre intervention) vaccine intentions in the video group and post intervention intentions in the control group (n=2089)

| **Study group** | **Non-intenders** n(%) | **Intenders** n(%) | **Total** n(%) |
| --- | --- | --- | --- |
| Active control (text) | 842 (84.7) | 152 (15.3) | 994 (47.6) |
| Video | 933 (85.2) | 162 (14.8) | 1095 (52.4) |
| Total | 1775 | 314 | 2089 |

X-squared=.10086, df=1, *P*=.7508

**Exploratory between-group analyses on the dataset that does not include careless responders (N=1373)**

**Table S10.** Baseline (pre intervention) vaccine intentions in the control group and post intervention intentions in the video group (N=1373)

| **Study group** | **Non-intenders** n(%) | **Intenders** n(%) | **Total** n(%) |
| --- | --- | --- | --- |
| Active control (text) | 600 (87.3) | 87 (12.7) | 687 (50.0) |
| Video | 567 (82.7) | 119 (17.3) | 686 (50.0) |
| Total | 1167 | 206 | 1373 |

X-squared=5.9033, df=1, *P*=.01511

**Table S11.** Baseline (pre intervention) vaccine intentions in the video group and post intervention intentions in the control group (N=1373)

| **Study group** | **Non-intenders** n(%) | **Intenders** n(%) | **Total** n(%) |
| --- | --- | --- | --- |
| Active control (text) | 581 (84.6) | 106 (15.4) | 687 (50.0) |
| Video | 600 (87.5) | 86 (12.5) | 686 (50.0) |
| Total | 1181 | 192 | 1373 |

X-squared=2.3883, df=1, *P*=.1222
