# Supplementary material for: The Efficacy of a Brief, Altruism-Eliciting Video Intervention in Enhancing COVID-19 Vaccination Intentions Among a Population-Based Sample of Younger Adults: Randomized Controlled Trial
Source: JMIR Public Health Surveill. 2022 May 30;8(5):e37328. doi: 10.2196/37328 (PMC9153910; doi:10.2196/37328)
Supplement: Multimedia Appendix 6 [file publichealth_v8i5e37328_app6.pdf]

CONSORT-EHEALTH (V 1.6.1) - Submission/Publication Form

The CONSORT-EHEALTH checklist is intended for authors of randomized trials evaluating web-based and Internet-based applications/interventions, including mobile interventions, electronic games (incl multiplayer games), social media, certain telehealth applications, and other interactive and/or networked electronic applications. Some of the items (e.g. all subitems under item 5 - description of the intervention) may also be applicable for other study designs.

The goal of the CONSORT EHEALTH checklist and guideline is to be
a) a guide for reporting for authors of RCTs,
b) to form a basis for appraisal of an ehealth trial (in terms of validity)

CONSORT-EHEALTH items/subitems are MANDATORY reporting items for studies published in the Journal of Medical Internet Research and other journals / scientific societies endorsing the checklist.

Items numbered 1., 2., 3., 4a., 4b etc are original CONSORT or CONSORT-NPT (non-pharmacologic treatment) items.
Items with Roman numerals (i., ii, iii, iv etc.) are CONSORT-EHEALTH extensions/clarifications.

As the CONSORT-EHEALTH checklist is still considered in a formative stage, we would ask that you also RATE ON A SCALE OF 1-5 how important/useful you feel each item is FOR THE PURPOSE OF THE CHECKLIST and reporting guideline (optional).

Mandatory reporting items are marked with a red *.
In the textboxes, either copy & paste the relevant sections from your manuscript into this form - please include any quotes from your manuscript in QUOTATION MARKS,
or answer directly by providing additional information not in the manuscript, or elaborating on why the item was not relevant for this study.

YOUR ANSWERS WILL BE PUBLISHED AS A SUPPLEMENTARY FILE TO YOUR PUBLICATION IN JMIR AND ARE CONSIDERED PART OF YOUR PUBLICATION (IF ACCEPTED).
Please fill in these questions diligently. Information will not be copyedited, so please use proper spelling and grammar, use correct capitalization, and avoid abbreviations.

DO NOT FORGET TO SAVE AS PDF _AND_ CLICK THE SUBMIT BUTTON SO YOUR ANSWERS ARE IN OUR DATABASE !!!

Citation Suggestion (if you append the pdf as Appendix we suggest to cite this paper in the caption):
Eysenbach G, CONSORT-EHEALTH Group
CONSORT-EHEALTH: Improving and Standardizing Evaluation Reports of Web-based and Mobile Health Interventions
J Med Internet Res 2011;13(4):e126
URL: http://www.jmir.org/2011/4/e126/
doi: 10.2196/jmir.1923
PMID: 22209829

benhaward1@gmail.com (not shared) Switch account
Draft saved

* Required

Your name *
First Last

Ovidiu Tatar

Primary Affiliation (short), City, Country *
University of Toronto, Toronto, Canada

Universite de Montreal, Montreal, Canada

Your e-mail address *
abc@gmail.com

Ovidiu.teofil.tatar@umontreal.ca

Title of your manuscript *
Provide the (draft) title of your manuscript.

Efficacy of a brief, altruism-eliciting video intervention in enhancing COVID-19 vaccination intentions amongst a population-based sample of younger adults: Randomized controlled trial.

Name of your App/Software/Intervention *
If there is a short and a long/alternate name, write the short name first and add the long name in brackets.

Altruism-Eliciting Video to Increase Vaccine In

Evaluated Version (if any)
e.g. "V1", "Release 2017-03-01", "Version 2.0.27913"

Release 2022-04-28

Language(s) *
What language is the intervention/app in? If multiple languages are available, separate by comma (e.g. "English, French")

English, French

URL of your Intervention Website or App
e.g. a direct link to the mobile app on app in appstore (itunes, Google Play), or URL of the website. If the intervention is a DVD or hardware, you can also link to an Amazon page.

https://www.youtube.com/watch?v=hNhNiingRjw&ab_channel=PSOLab

URL of an image/screenshot (optional)

Your answer

Accessibility *
Can an enduser access the intervention presently?

- (●) access is free and open
- ( ) access only for special usergroups, not open
- ( ) access is open to everyone, but requires payment/subscription/in-app purchases
- ( ) app/intervention no longer accessible
- ( ) Other:

Primary Medical Indication/Disease/Condition *
e.g. "Stress", "Diabetes", or define the target group in brackets after the condition, e.g. "Autism (Parents of children with)", "Alzheimers (Informal Caregivers of)"

Vaccine hesitancy

Primary Outcomes measured in trial *
comma-separated list of primary outcomes reported in the trial

COVID-19 vaccine intentions (measured using

Secondary/other outcomes
Are there any other outcomes the intervention is expected to affect?

Your answer

Recommended "Dose" *
What do the instructions for users say on how often the app should be used?

- ( ) Approximately Daily
- ( ) Approximately Weekly
- ( ) Approximately Monthly
- ( ) Approximately Yearly
- (●) "as needed"
- ( ) Other:

Approx. Percentage of Users (starters) still using the app as recommended after 3 months *

- (●) unknown / not evaluated
- ( ) 0-10%
- ( ) 11-20%
- ( ) 21-30%
- ( ) 31-40%
- ( ) 41-50%
- ( ) 51-60%
- ( ) 61-70%
- ( ) 71%-80%
- ( ) 81-90%
- ( ) 91-100%
- ( ) Other:

Overall, was the app/intervention effective? *

- ( ) yes: all primary outcomes were significantly better in intervention group vs control
- (●) partly: SOME primary outcomes were significantly better in intervention group vs control
- ( ) no statistically significant difference between control and intervention
- ( ) potentially harmful: control was significantly better than intervention in one or more outcomes
- ( ) inconclusive: more research is needed
- ( ) Other:

Article Preparation Status/Stage *
At which stage in your article preparation are you currently (at the time you fill in this form)

- ( ) not submitted yet - in early draft status
- ( ) not submitted yet - in late draft status, just before submission
- ( ) submitted to a journal but not reviewed yet
- ( ) submitted to a journal and after receiving initial reviewer comments
- ( ) submitted to a journal and accepted, but not published yet
- ( ) published
- (●) Other: Accepted by JMIR Public Health and Surveillance

Journal *
If you already know where you will submit this paper (or if it is already submitted), please provide the journal name (if it is not JMIR, provide the journal name under "other")

- ( ) not submitted yet / unclear where I will submit this
- ( ) Journal of Medical Internet Research (JMIR)
- ( ) JMIR mHealth and UHealth
- ( ) JMIR Serious Games
- ( ) JMIR Mental Health
- (●) JMIR Public Health
- ( ) JMIR Formative Research
- ( ) Other JMIR sister journal
- ( ) Other:

Is this a full powered effectiveness trial or a pilot/feasibility trial? *

- ( ) Pilot/feasibility
- (●) Fully powered

Manuscript tracking number *
If this is a JMIR submission, please provide the manuscript tracking number under "other" (The ms tracking number can be found in the submission acknowledgement email, or when you login as author in JMIR. If the paper is already published in JMIR, then the ms tracking number is the four-digit number at the end of the DOI, to be found at the bottom of each published article in JMIR)

- ( ) no ms number (yet) / not (yet) submitted to / published in JMIR
- (●) Other: ms#37328

TITLE AND ABSTRACT

1a) TITLE: Identification as a randomized trial in the title

1a) Does your paper address CONSORT item 1a? *
I.e does the title contain the phrase "Randomized Controlled Trial"? (if not, explain the reason under "other")

- (●) yes
- ( ) Other:

1a-i) Identify the mode of delivery in the title
Identify the mode of delivery. Preferably use "web-based" and/or "mobile" and/or "electronic game" in the title. Avoid ambiguous terms like "online", "virtual", "interactive". Use "Internet-based" only if Intervention includes non-web-based Internet components (e.g. email), use "computer-based" or "electronic" only if offline products are used. Use "virtual" only in the context of "virtual reality" (3-D worlds). Use "online" only in the context of "online support groups". Complement or substitute product names with broader terms for the class of products (such as "mobile" or "smart phone" instead of "iphone"), especially if the application runs on different platforms.

subitem not at all important 1 ( ) 2 ( ) 3 ( ) 4 (●) 5 ( ) essential
Clear selection

Does your paper address subitem 1a-i? *
Copy and paste relevant sections from manuscript title (include quotes in quotation marks "like this" to indicate direct quotes from your manuscript), or elaborate on this item by providing additional information not in the ms, or briefly explain why the item is not applicable/relevant for your study

Mode of delivery is not included In the title, but it is detailed that a video intervention is tested, implying an electronically delivered intervention.

1a-ii) Non-web-based components or important co-interventions in title
Mention non-web-based components or important co-interventions in title, if any (e.g., "with telephone support").

subitem not at all important 1 ( ) 2 (●) 3 ( ) 4 ( ) 5 ( ) essential
Clear selection

Does your paper address subitem 1a-ii?
Copy and paste relevant sections from manuscript title (include quotes in quotation marks "like this" to indicate direct quotes from your manuscript), or elaborate on this item by providing additional information not in the ms, or briefly explain why the item is not applicable/relevant for your study

N/A - no non-web-based components in the study.

1a-iii) Primary condition or target group in the title
Mention primary condition or target group in the title, if any (e.g., "for children with Type I Diabetes") Example: A Web-based and Mobile Intervention with Telephone Support for Children with Type I Diabetes: Randomized Controlled Trial

subitem not at all important 1 ( ) 2 ( ) 3 ( ) 4 (●) 5 ( ) essential
Clear selection

Does your paper address subitem 1a-iii? *
Copy and paste relevant sections from manuscript title (include quotes in quotation marks "like this" to indicate direct quotes from your manuscript), or elaborate on this item by providing additional information not in the ms, or briefly explain why the item is not applicable/relevant for your study

"enhancing COVID-19 vaccination intentions amongst a population-based sample of younger adults"

1b) ABSTRACT: Structured summary of trial design, methods, results, and conclusions
NPT extension: Description of experimental treatment, comparator, care providers, centers, and blinding status.

1b-i) Key features/functionalities/components of the intervention and comparator in the METHODS section of the ABSTRACT
Mention key features/functionalities/components of the intervention and comparator in the abstract. If possible, also mention theories and principles used for designing the site. Keep in mind the needs of systematic reviewers and indexers by including important synonyms. (Note: Only report in the abstract what the main paper is reporting. If this information is missing from the main body of text, consider adding it)

subitem not at all important 1 ( ) 2 ( ) 3 ( ) 4 ( ) 5 (●) essential
Clear selection

Does your paper address subitem 1b-i? *
Copy and paste relevant sections from the manuscript abstract (include quotes in quotation marks "like this" to indicate direct quotes from your manuscript), or elaborate on this item by providing additional information not in the ms, or briefly explain why the item is not applicable/relevant for your study

"This study had two objectives: (1) to evaluate the within-group efficacy of an altruism-eliciting video intervention in increasing COVID-19 vaccination intentions amongst younger adults; and (2) to examine the video's efficacy compared to a text-based intervention focused exclusively on non- vaccine related COVID-19 preventive health measures."

1b-ii) Level of human involvement in the METHODS section of the ABSTRACT
Clarify the level of human involvement in the abstract, e.g., use phrases like "fully automated" vs. "therapist/nurse/care provider/physician-assisted" (mention number and expertise of providers involved, if any). (Note: Only report in the abstract what the main paper is reporting. If this information is missing from the main body of text, consider adding it)

subitem not at all important 1 (●) 2 ( ) 3 ( ) 4 ( ) 5 ( ) essential
Clear selection

Does your paper address subitem 1b-ii?
Copy and paste relevant sections from the manuscript abstract (include quotes in quotation marks "like this" to indicate direct quotes from your manuscript), or elaborate on this item by providing additional information not in the ms, or briefly explain why the item is not applicable/relevant for your study

N/A - web-based survey using video-based and text-based interventions.

1b-iii) Open vs. closed, web-based (self-assessment) vs. face-to-face assessments in the METHODS section of the ABSTRACT
Mention how participants were recruited (online vs. offline), e.g., from an open access website or from a clinic or a closed online user group (closed usergroup trial), and clarify if this was a purely web-based trial, or there were face-to-face components (as part of the intervention or for assessment). Clearly say if outcomes were self-assessed through questionnaires (as common in web-based trials). Note: In traditional offline trials, an open trial (open-label trial) is a type of clinical trial in which both the researchers and participants know which treatment is being administered. To avoid confusion, use "blinded" or "unblinded" to indicated the level of blinding instead of "open", as "open" in web-based trials usually refers to "open access" (i.e. participants can self-enrol). (Note: Only report in the abstract what the main paper is reporting. If this information is missing from the main body of text, consider adding it)

subitem not at all important 1 ( ) 2 ( ) 3 ( ) 4 (●) 5 ( ) essential
Clear selection

Does your paper address subitem 1b-iii?
Copy and paste relevant sections from the manuscript abstract (include quotes in quotation marks "like this" to indicate direct quotes from your manuscript), or elaborate on this item by providing additional information not in the ms, or briefly explain why the item is not applicable/relevant for your study

"Using a web-based survey in a pre-to-post, randomized control trial design, we recruited Canadians aged 20-39 who were not yet vaccinated against COVID-19 from an online survey panel and randomized them in a 1:1 ratio to receive either the video intervention or an active text control."

1b-iv) RESULTS section in abstract must contain use data
Report number of participants enrolled/assessed in each group, the use/uptake of the intervention (e.g., attrition/adherence metrics, use over time, number of logins etc.), in addition to primary/secondary outcomes. (Note: Only report in the abstract what the main paper is reporting. If this information is missing from the main body of text, consider adding it)

subitem not at all important 1 ( ) 2 ( ) 3 ( ) 4 ( ) 5 (●) essential
Clear selection

Does your paper address subitem 1b-iv?
Copy and paste relevant sections from the manuscript abstract (include quotes in quotation marks "like this" to indicate direct quotes from your manuscript), or elaborate on this item by providing additional information not in the ms, or briefly explain why the item is not applicable/relevant for your study

"Analyses were performed on N = 1373 participants (n = 686 in video arm, n = 687 in text arm)."

"Within-group results for the video intervention arm showed that there was a significant change on intentions to receive the vaccine ($\chi2$ (1) = 20.55, P < .001). The between-group difference in post- intervention intentions ($\chi2$ (3) = 1.70, P = .64) was not significant. When administered the video intervention, we found that participants who had not thought about or were undecided about receiving a COVID-19 vaccine were more amenable to change than participants who had already decided not to vaccinate."

1b-v) CONCLUSIONS/DISCUSSION in abstract for negative trials
Conclusions/Discussions in abstract for negative trials: Discuss the primary outcome - if the trial is negative (primary outcome not changed), and the intervention was not used, discuss whether negative results are attributable to lack of uptake and discuss reasons. (Note: Only report in the abstract what the main paper is reporting. If this information is missing from the main body of text, consider adding it)

subitem not at all important 1 ( ) 2 ( ) 3 ( ) 4 ( ) 5 (●) essential
Clear selection

Does your paper address subitem 1b-v?
Copy and paste relevant sections from the manuscript abstract (include quotes in quotation marks "like this" to indicate direct quotes from your manuscript), or elaborate on this item by providing additional information not in the ms, or briefly explain why the item is not applicable/relevant for your study

"While the video intervention was limited in its effect on those who had firmly decided not to vaccinate, our study demonstrates that prosocial and altruistic messages could increase COVID-19 vaccine uptake, especially when targeted to younger adults who are undecided or unengaged regarding vaccination. This might indicate that altruistic messaging provides a "push" for those who are tentative towards, or removed from, the decision to receive the vaccine."

INTRODUCTION

2a) In INTRODUCTION: Scientific background and explanation of rationale

2a-i) Problem and the type of system/solution
Describe the problem and the type of system/solution that is object of the study: intended as stand-alone intervention vs. incorporated in broader health care program? Intended for a particular patient population? Goals of the intervention, e.g., being more cost-effective to other interventions, replace or complement other solutions? (Note: Details about the intervention are provided in "Methods" under 5)

subitem not at all important 1 ( ) 2 ( ) 3 ( ) 4 ( ) 5 (●) essential
Clear selection

Does your paper address subitem 2a-i? *
Copy and paste relevant sections from the manuscript (include quotes in quotation marks "like this" to indicate direct quotes from your manuscript), or elaborate on this item by providing additional information not in the ms, or briefly explain why the item is not applicable/relevant for your study

"Vaccine hesitancy, which refers to a set of attitudes and beliefs which may lead to delay or refusal of one or more vaccines despite their availability [8, 9], poses a significant threat to achieving sufficient COVID-19 vaccination rates to mitigate the pandemic. Younger age has been associated with vaccine hesitancy [10-14]. Additionally, younger adults often experience mild or asymptomatic infections [15, 16], and are more socially active. In Canada, this age group also demonstrates lower adherence to other preventive health measures (e.g., social distancing) [17, 18]. Thus, younger adults play an important role in virus transmission. To protect the Canadian population at large, it is important to ensure adequate vaccine uptake amongst younger adults."

"Considering the need to address hesitancy towards COVID-19 vaccination amongst younger adults, the aim of this study was to evaluate the efficacy of a short video intervention eliciting altruistic motives for vaccination."

2a-ii) Scientific background, rationale: What is known about the (type of) system
Scientific background, rationale: What is known about the (type of) system that is the object of the study (be sure to discuss the use of similar systems for other conditions/diagnoses, if appropiate), motivation for the study, i.e. what are the reasons for and what is the context for this specific study, from which stakeholder viewpoint is the study performed, potential impact of findings [2]. Briefly justify the choice of the comparator.

subitem not at all important 1 ( ) 2 ( ) 3 ( ) 4 (●) 5 ( ) essential
Clear selection

Does your paper address subitem 2a-ii? *
Copy and paste relevant sections from the manuscript (include quotes in quotation marks "like this" to indicate direct quotes from your manuscript), or elaborate on this item by providing additional information not in the ms, or briefly explain why the item is not applicable/relevant for your study

"A novel and promising approach is to develop interventions that elicit altruism, i.e., intentional and voluntary action in which the primary goal is to increase the welfare of another person [23, 24]. Previous hypothetical and laboratory game studies have found that altruistic messages can increase vaccination intentions [25-27] or demonstrated that altruistic motives were related to self-reports of actual vaccine intentions and/or behaviors. However, few studies have experimentally elicited altruism to examine its impact on vaccine intentions [28, 29], and none have used a video-based intervention. Younger adults have lower concerns of hospitalization and mortality than older adults [30], and thus may perceive receiving a COVID-19 vaccine as less personally beneficial. To increase vaccination intentions and uptake amongst this age group, it could be more effective to highlight messages of altruism and the protection of others rather than oneself [12, 31]."

The choice of the control group is described in the methods under the "interventions" heading: "Consistent with the widespread use of public health messaging campaigns during the pandemic focusing on promoting preventive health behaviours, we decided to include an active instead of a placebo control group. We developed the text-based intervention by selecting non- vaccine related preventive health behaviour recommendations disseminated through the Public Health Agency of Canada's website [47]."

2b) In INTRODUCTION: Specific objectives or hypotheses

Does your paper address CONSORT subitem 2b? *
Copy and paste relevant sections from the manuscript (include quotes in quotation marks "like this" to indicate direct quotes from your manuscript), or elaborate on this item by providing additional information not in the ms, or briefly explain why the item is not applicable/relevant for your study

"The specific objectives were to estimate: (1) pre- to post-intervention change of COVID-19 vaccine intentions, and (2) between-group COVID-19 vaccine intentions post-intervention."

METHODS

3a) Description of trial design (such as parallel, factorial) including allocation ratio

Does your paper address CONSORT subitem 3a? *
Copy and paste relevant sections from the manuscript (include quotes in quotation marks "like this" to indicate direct quotes from your manuscript), or elaborate on this item by providing additional information not in the ms, or briefly explain why the item is not applicable/relevant for your study

"We used a two-arm parallel randomized pre-post design. Participants in a web-based survey were randomly allocated in a 1:1 ratio to the video-based intervention or the active control arm consisting of a text-based intervention."

3b) Important changes to methods after trial commencement (such as eligibility criteria), with reasons

Does your paper address CONSORT subitem 3b? *
Copy and paste relevant sections from the manuscript (include quotes in quotation marks "like this" to indicate direct quotes from your manuscript), or elaborate on this item by providing additional information not in the ms, or briefly explain why the item is not applicable/relevant for your study

N/A - no changes to study methods after trial commencement

3b-i) Bug fixes, Downtimes, Content Changes
Bug fixes, Downtimes, Content Changes: ehealth systems are often dynamic systems. A description of [illegible]

changes to methods therefore also includes important changes made on the intervention or comparator during the trial (e.g., major bug fixes or changes in the functionality or content) (5-iii) and other "unexpected events" that may have influenced study design such as staff changes, system failures/downtimes, etc. [2].

	1	2	3	4	5	
subitem not at all important	◉	○	○	○	○	essential

Clear selection

Does your paper address subitem 3b-i?
Copy and paste relevant sections from the manuscript (include quotes in quotation marks "like this" to indicate direct quotes from your manuscript), or elaborate on this item by providing additional information not in the ms, or briefly explain why the item is not applicable/relevant for your study

N/A - no bug fixes, downtime, content changes

4a) Eligibility criteria for participants

Does your paper address CONSORT subitem 4a? *
Copy and paste relevant sections from the manuscript (include quotes in quotation marks "like this" to indicate direct quotes from your manuscript), or elaborate on this item by providing additional information not in the ms, or briefly explain why the item is not applicable/relevant for your study

"Participants from all Canadian provinces or territories who met following eligibility criteria were enrolled in the study: (1) not vaccinated for COVID-19; (2) age range of 20 to 39 years; (3) Canadian resident; and (4) willing to complete the survey in either English or French"

4a-i) Computer / Internet literacy
Computer / Internet literacy is often an implicit "de facto" eligibility criterion - this should be explicitly clarified.

	1	2	3	4	5	
subitem not at all important	◉	○	○	○	○	essential

Clear selection

Does your paper address subitem 4a-i?
Copy and paste relevant sections from the manuscript (include quotes in quotation marks "like this" to indicate direct quotes from your manuscript), or elaborate on this item by providing additional information not in the ms, or briefly explain why the item is not applicable/relevant for your study

"Dynata used a combination of recruitment methods (e.g., their own website, direct emails, ads on social media) to recruit participants from their Canadian survey panel"

Considering participants are on an online survey panel and that the study population was younger adults (aged 20-39), computer and internet literacy were implied.

4a-ii) Open vs. closed, web-based vs. face-to-face assessments:
Open vs. closed, web-based vs. face-to-face assessments: Mention how participants were recruited (online vs. offline), e.g., from an open access website or from a clinic, and clarify if this was a purely web-based trial, or there were face-to-face components (as part of the intervention or for assessment), i.e., to what degree got the study team to know the participant. In online-only trials, clarify if participants were quasi-anonymous and whether having multiple identities was possible or whether technical or logistical measures (e.g., cookies, email confirmation, phone calls) were used to detect/prevent these.

	1	2	3	4	5	
subitem not at all important	○	○	○	◉	○	essential

Clear selection

Does your paper address subitem 4a-ii? *
Copy and paste relevant sections from the manuscript (include quotes in quotation marks "like this" to indicate direct quotes from your manuscript), or elaborate on this item by providing additional information not in the ms, or briefly explain why the item is not applicable/relevant for your study

The trial was purely web-based and participants were recruited from Dynata's panel of Canadian residents. This panel recruits using a "by-invitation-only" method where participants' identities are validated by other partnering companies. All data collection was anonymous but participants have unique IDs assigned to them by Dynata which can be used to detect duplicate responses as well as a check for duplicate IP addresses.

4a-iii) Information giving during recruitment
Information given during recruitment. Specify how participants were briefed for recruitment and in the informed consent procedures (e.g., publish the informed consent documentation as appendix, see also item X26), as this information may have an effect on user self-selection, user expectation and may also bias results.

	1	2	3	4	5	
subitem not at all important	○	○	○	◉	○	essential

Clear selection

Does your paper address subitem 4a-iii?
Copy and paste relevant sections from the manuscript (include quotes in quotation marks "like this" to indicate direct quotes from your manuscript), or elaborate on this item by providing additional information not in the ms, or briefly explain why the item is not applicable/relevant for your study

Participants were invited to complete a survey about "Health and Wellness". In the informed consent form, participants were informed they would either watch a video or read a text about the COVID-19 pandemic during the study.

4b) Settings and locations where the data were collected

Does your paper address CONSORT subitem 4b? *
Copy and paste relevant sections from the manuscript (include quotes in quotation marks "like this" to indicate direct quotes from your manuscript), or elaborate on this item by providing additional information not in the ms, or briefly explain why the item is not applicable/relevant for your study

"Participants in a web-based survey were randomly allocated in a 1:1 ratio to the video-based intervention or the active control arm consisting of a text-based intervention."

"Dynata used a combination of recruitment methods (e.g., their own website, direct emails, ads on social media) to recruit participants."

4b-i) Report if outcomes were (self-)assessed through online questionnaires
Clearly report if outcomes were (self-)assessed through online questionnaires (as common in web-based trials) or otherwise.

	1	2	3	4	5	
subitem not at all important	○	◉	○	○	○	essential

Clear selection

Does your paper address subitem 4b-i? *
Copy and paste relevant sections from the manuscript (include quotes in quotation marks "like this" to indicate direct quotes from your manuscript), or elaborate on this item by providing additional information not in the ms, or briefly explain why the item is not applicable/relevant for your study

All measures were self-assessed as it was a fully autonomous web-based survey.

4b-ii) Report how institutional affiliations are displayed
Report how institutional affiliations are displayed to potential participants [on ehealth media], as affiliations with prestigious hospitals or universities may affect volunteer rates, use, and reactions with regards to an intervention.(Not a required item – describe only if this may bias results)

	1	2	3	4	5	
subitem not at all important	◉	○	○	○	○	essential

Clear selection

Does your paper address subitem 4b-ii?
Copy and paste relevant sections from the manuscript (include quotes in quotation marks "like this" to indicate direct quotes from your manuscript), or elaborate on this item by providing additional information not in the ms, or briefly explain why the item is not applicable/relevant for your study

N/A - affiliations were only provided in the consent form to enable contact with the principal investigator (ZR) and are unlikely to have biased results.

5) The interventions for each group with sufficient details to allow replication, including how and when they were actually administered

5-i) Mention names, credential, affiliations of the developers, sponsors, and owners
Mention names, credential, affiliations of the developers, sponsors, and owners [6] (if authors/evaluators are owners or developer of the software, this needs to be declared in a "Conflict of interest" section or mentioned elsewhere in the manuscript).

	1	2	3	4	5	
subitem not at all important	○	◉	○	○	○	essential

Clear selection

Does your paper address subitem 5-i?
Copy and paste relevant sections from the manuscript (include quotes in quotation marks "like this" to indicate direct quotes from your manuscript), or elaborate on this item by providing additional information not in the ms, or briefly explain why the item is not applicable/relevant for your study

"The development of the intervention unfolded in the following phases: first, we developed the script to focus on three characters with different COVID-19 vulnerability profiles... Subsequently, an initial storyboard was created by Akufen (a Montreal-based media design company) which was further refined and produced in video format."

The principal investigator (ZR) owns the final video but it is not being used directly for any purpose other than this study at this moment.

5-ii) Describe the history/development process
Describe the history/development process of the application and previous formative evaluations (e.g., focus groups, usability testing), as these will have an impact on adoption/use rates and help with interpreting results.

	1	2	3	4	5	
subitem not at all important	○	○	○	◉	○	essential

Clear selection

Does your paper address subitem 5-ii?
Copy and paste relevant sections from the manuscript (include quotes in quotation marks "like this" to indicate direct quotes from your manuscript), or elaborate on this item by providing additional information not in the ms, or briefly explain why the item is not applicable/relevant for your study

"Adjustments were made based on the feedback received from five young adults (aged 20-39 who had not yet received the COVID-19 vaccine) who viewed the video and participated a focus group in June 2021."

The video was refined based on these focus groups to produce the final video used in this study.

5-iii) Revisions and updating
Revisions and updating. Clearly mention the date and/or version number of the application/intervention (and comparator, if applicable) evaluated, or describe whether the intervention underwent major changes during the evaluation process, or whether the development and/or content was "frozen" during the trial. Describe dynamic components such as news feeds or changing content which may have an impact on the replicability of the intervention (for unexpected events see item 3b).

	1	2	3	4	5	
subitem not at all important	○	○	○	◉	○	essential

Clear selection

Does your paper address subitem 5-iii?
Copy and paste relevant sections from the manuscript (include quotes in quotation marks "like this" to indicate direct quotes from your manuscript), or elaborate on this item by providing additional information not in the ms, or briefly explain why the item is not applicable/relevant for your study

"Adjustments were made based on the feedback received from five young adults (aged 20-39 who had not yet received the COVID-19 vaccine) who viewed the video and participated a focus group in June 2021. The final animated character video was two minutes and forty-seven seconds in length."

The intervention did not change after the beginning of the trial.

5-iv) Quality assurance methods
Provide information on quality assurance methods to ensure accuracy and quality of information provided [1], if applicable.

	1	2	3	4	5	
subitem not at all important	○	○	○	◉	○	essential

Clear selection

Does your paper address subitem 5-iv?
Copy and paste relevant sections from the manuscript (include quotes in quotation marks "like this" to indicate direct quotes from your manuscript), or elaborate on this item by providing additional information not in the ms, or briefly explain why the item is not applicable/relevant for your study

"The development of the intervention was informed by a literature review conducted by our team showing that eliciting prosocial motives (altruism) can increase vaccine intentions. Accordingly, the messaging was framed around the concept of social benefit of vaccination by emphasizing the importance of indirectly protecting the health of vulnerable individuals who either cannot receive the vaccine (e.g., children under the age of five) or who might develop an insufficient immune response (e.g. elderly, immunocompromised) [36-41]."

"We developed the text-based intervention by selecting non-vaccine related preventive health behaviour recommendations disseminated through the Public Health Agency of Canada's website [47]. The text-based intervention was limited to about 450 words to ensure a reading time similar to the duration of the video-based intervention. Recommendations were divided in three sections: travel restrictions (e.g., mandatory COVID-19 testing, mandatory isolation), general hygiene (e.g., handwashing, mask-wearing), and physical distancing (e.g., avoiding closed spaces, maintaining a physical distance of 2 meters from people outside of your household)."

The video was also shared with research collaborators (GZ, ED, MT) to check for quality and applicability to COVID-19 vaccination.

5-v) Ensure replicability by publishing the source code, and/or providing screenshots/screen-capture video, and/or providing flowcharts of the algorithms used
Ensure replicability by publishing the source code, and/or providing screenshots/screen-capture video, and/or providing flowcharts of the algorithms used. Replicability (i.e., other researchers should in principle be able to replicate the study) is a hallmark of scientific reporting.

	1	2	3	4	5	
subitem not at all important	○	○	○	◉	○	essential

Clear selection

Does your paper address subitem 5-v?
Copy and paste relevant sections from the manuscript (include quotes in quotation marks "like this" to indicate direct quotes from your manuscript), or elaborate on this item by providing additional information not in the ms, or briefly explain why the item is not applicable/relevant for your study

"(Click to view the videos in English [45] or French [46])."

Video links are available in the references.

5-vi) Digital preservation
Digital preservation: Provide the URL of the application, but as the intervention is likely to change or disappear over the course of the years; also make sure the intervention is archived (Internet Archive, webcitation.org, and/or publishing the source code or screenshots/videos alongside the article). As pages behind login screens cannot be archived, consider creating demo pages which are accessible without login.

	1	2	3	4	5	
subitem not at all important	○	○	○	◉	○	essential

Clear selection

Does your paper address subitem 5-vi?
Copy and paste relevant sections from the manuscript (include quotes in quotation marks "like this" to indicate direct quotes from your manuscript), or elaborate on this item by providing additional information not in the ms, or briefly explain why the item is not applicable/relevant for your study

"(Click to view the videos in English [45] or French [46])."

Video links are available in the references.

5-vii) Access
Access: Describe how participants accessed the application, in what setting/context, if they had to pay (or were paid) or not, whether they had to be a member of specific group. If known, describe how participants obtained "access to the platform and Internet" [1]. To ensure access for editors/reviewers/readers, consider to provide a "backdoor" login account or demo mode for reviewers/readers to explore the application (also important for archiving purposes, see vi).

	1	2	3	4	5	
subitem not at all important	○	○	○	◉	○	essential

Clear selection

Does your paper address subitem 5-vii? *
Copy and paste relevant sections from the manuscript (include quotes in quotation marks "like this" to indicate direct quotes from your manuscript), or elaborate on this item by providing additional information not in the ms, or briefly explain why the item is not applicable/relevant for your study

Dynata's survey panelists are recruited through a "By-Invitation-Only" method, in which their identities are validated by partnered businesses to ensure response quality and validity. For our study, Dynata used a combination of recruitment methods (e.g., their own website, direct emails, ads on social media). Participants were compensated using Dynata's rewards program, in which they can accumulate points to redeem at partnered businesses (e.g., Starbucks, Amazon).

5-viii) Mode of delivery, features/functionalities/components of the intervention and comparator, and the theoretical framework
Describe mode of delivery, features/functionalities/components of the intervention and comparator, and the theoretical framework [6] used to design them (instructional strategy [1], behaviour change techniques, persuasive features, etc., see e.g., [7, 8] for terminology). This includes an in-depth description of the content (including where it is coming from and who developed it) [1]," whether [and how] it is tailored to individual circumstances and allows users to track their progress and receive feedback" [6]. This also includes a description of communication delivery channels and – if computer-mediated communication is a component – whether communication was synchronous or asynchronous [6]. It also includes information on presentation strategies [1], including page design principles, average amount of text on pages, presence of hyperlinks to other resources, etc. [1].

	1	2	3	4	5	
subitem not at all important	○	○	○	○	○	essential

Does your paper address subitem 5-viii? *
Copy and paste relevant sections from the manuscript (include quotes in quotation marks "like this" to indicate direct quotes from your manuscript), or elaborate on this item by providing additional information not in the ms, or briefly explain why the item is not applicable/relevant for your study

"Video intervention.

Because mobile streaming is highly popular in our target age-group [35], we decided to use a video-based intervention to maximize its acceptability and minimize study attrition. The development of the intervention was informed by a literature review conducted by our team showing that eliciting prosocial motives (altruism) can increase vaccine intentions. Accordingly, the messaging was framed around the concept of social benefit of vaccination by emphasizing the importance of indirectly protecting the health of vulnerable individuals who either cannot receive the vaccine (e.g., children under the age of five) or who might develop an insufficient immune response (e.g. elderly, immunocompromised) [36-41]. Moreover, protecting children and elderly and providing details about negative health outcomes caused by infection were found to elicit empathy and altruism and increase vaccine acceptability in young adults [28, 42, 43]. Because narratives represent an essential component of human communication and their use has been recommended for health behavior change interventions [44], we used this approach to emphasize the importance of receiving the vaccine for protecting others. Finally, we drew a parallel between the collective benefits of having a public health system and the social benefits of being adequately vaccinated.

The development of the intervention unfolded in the following phases: first, we developed the script to focus on three characters with different COVID-19 vulnerability profiles i.e., John, 82 years old, vaccinated but at risk because of his age; Simon, 4 years old, not eligible for vaccination at the time of the study; and Marie, 32 years old, at risk of infection because of the immunosuppressive effects of chemotherapy. Subsequently, an initial storyboard was created by Akufen (a Montreal-based media design company) which was further refined and produced in video format. Adjustments were made based on the feedback received from five young adults (aged 20-39 who had not yet received the COVID-19 vaccine) who viewed the video and participated a focus group in June 2021. The final animated character video was two minutes and forty-seven seconds in length. (Click to view the videos in English [45] or French [46]). All narration was completed by an experienced, fully bilingual professional narrator.

Text intervention.

Consistent with the widespread use of public health messaging campaigns during the pandemic focusing on promoting preventive health behaviours, we decided to include an active instead of a placebo control group. We developed the text-based intervention by selecting non-vaccine related preventive health behaviour recommendations disseminated through the Public Health Agency of Canada's website [47]. The text-based intervention was limited to about 450 words to ensure a reading time similar to the duration of the video-based intervention. Recommendations were divided in three sections: travel restrictions (e.g., mandatory COVID-19 testing, mandatory isolation), general hygiene (e.g., handwashing, mask-wearing), and physical distancing (e.g., avoiding closed spaces, maintaining a physical distance of 2 meters from people outside of your household). See Appendix C for the text intervention and attention check questions."

5-ix) Describe use parameters
Describe use parameters (e.g., intended "doses" and optimal timing for use). Clarify what instructions or recommendations were given to the user, e.g., regarding timing, frequency, heaviness of use, if any, or was the intervention used ad libitum.

	1	2	3	4	5	
subitem not at all important	○	◉	○	○	○	essential

Clear selection

Does your paper address subitem 5-ix?
Copy and paste relevant sections from the manuscript (include quotes in quotation marks "like this" to indicate direct quotes from your manuscript), or elaborate on this item by providing additional information not in the ms, or briefly explain why the item is not applicable/relevant for your study

N/A - parameters were not relevant for our interventions (watching a video or reading a text).

5-x) Clarify the level of human involvement
Clarify the level of human involvement (care providers or health professionals, also technical assistance) in the e-intervention or as co-intervention (detail number and expertise of professionals involved, if any, as well as "type of assistance offered, the timing and frequency of the support, how it is initiated, and the medium by which the assistance is delivered". It may be necessary to distinguish between the level of human involvement required for the trial, and the level of human involvement required for a routine application outside of a RCT setting (discuss under item 21 – generalizability).

	1	2	3	4	5	
subitem not at all important	○	◉	○	○	○	essential

Clear selection

Does your paper address subitem 5-x?
Copy and paste relevant sections from the manuscript (include quotes in quotation marks "like this" to indicate direct quotes from your manuscript), or elaborate on this item by providing additional information not in the ms, or briefly explain why the item is not applicable/relevant for your study

N/A - web-based survey using video-based and text-based interventions.

5-xi) Report any prompts/reminders used
Report any prompts/reminders used: Clarify if there were prompts (letters, emails, phone calls, SMS) to use the application, what triggered them, frequency etc. It may be necessary to distinguish between the level of prompts/reminders required for the trial, and the level of prompts/reminders for a routine application outside of a RCT setting (discuss under item 21 – generalizability).

	1	2	3	4	5	
subitem not at all important	○	◉	○	○	○	essential

Clear selection

Does your paper address subitem 5-xi? *
Copy and paste relevant sections from the manuscript (include quotes in quotation marks "like this" to indicate direct quotes from your manuscript), or elaborate on this item by providing additional information not in the ms, or briefly explain why the item is not applicable/relevant for your study

N/A - cross-sectional web-based survey

5-xii) Describe any co-interventions (incl. training/support)
Describe any co-interventions (incl. training/support): Clearly state any interventions that are provided in addition to the targeted eHealth intervention, as ehealth intervention may not be designed as stand-alone intervention. This includes training sessions and support [1]. It may be necessary to distinguish between the level of training required for the trial, and the level of training for a routine application outside of a RCT setting (discuss under item 21 – generalizability.

	1	2	3	4	5	
subitem not at all important	○	◉	○	○	○	essential

Clear selection

Does your paper address subitem 5-xii? *
Copy and paste relevant sections from the manuscript (include quotes in quotation marks "like this" to indicate direct quotes from your manuscript), or elaborate on this item by providing additional information not in the ms, or briefly explain why the item is not applicable/relevant for your study

N/A - no co-interventions

6a) Completely defined pre-specified primary and secondary outcome measures, including how and when they were assessed

Does your paper address CONSORT subitem 6a? *
Copy and paste relevant sections from the manuscript (include quotes in quotation marks "like this" to indicate direct quotes from your manuscript), or elaborate on this item by providing additional information not in the ms, or briefly explain why the item is not applicable/relevant for your study

"Therefore, to measure COVID-19 vaccine intentions we used a stage-based model of health decision-making, the Precaution Adoption Process Model (PAPM) [50]. Informed by the PAPM, we asked participants "Which of the following best describes your thoughts about a COVID-19 vaccine?" and allowed participants to place themselves in one of four nominal intention stages: (1) unengaged ("At this moment I have not thought about receiving the COVID-19 vaccine"); (2) undecided ("At this moment I am undecided about receiving the COVID-19 vaccine"); (3) decided not ("At this moment I do NOT want to receive the COVID-19 vaccine"); and (4) decided to ("At this moment I do want to receive the COVID-19 vaccine")."

6a-i) Online questionnaires: describe if they were validated for online use and apply CHERRIES items to describe how the questionnaires were designed/deployed
If outcomes were obtained through online questionnaires, describe if they were validated for online use and apply CHERRIES items to describe how the questionnaires were designed/deployed [9].

	1	2	3	4	5	
subitem not at all important	○	○	◉	○	○	essential

Clear selection

Does your paper address subitem 6a-i?
Copy and paste relevant sections from manuscript text

N/A - CHERRIES has not been applied to the given study questionnaire. The programmed questionnaire was tested extensively by the research team before data collection.

6a-ii) Describe whether and how "use" (including intensity of use/dosage) was defined/measured/monitored
Describe whether and how "use" (including intensity of use/dosage) was defined/measured/monitored (logins, logfile analysis, etc.). Use/adoption metrics are important process outcomes that should be reported in any ehealth trial.

	1	2	3	4	5	
subitem not at all important	○	○	◉	○	○	essential

Clear selection

Does your paper address subitem 6a-ii?
Copy and paste relevant sections from manuscript text

N/A - not applicable to the research design

6a-iii) Describe whether, how, and when qualitative feedback from participants was obtained
Describe whether, how, and when qualitative feedback from participants was obtained (e.g., through emails, feedback forms, interviews, focus groups).

	1	2	3	4	5	
subitem not at all important	○	○	○	◉	○	essential

Clear selection

Does your paper address subitem 6a-iii?
Copy and paste relevant sections from manuscript text

No qualitative feedback was obtained from the participants who completed the survey, but focus groups were used to refine the video intervention before the trial began.

"Adjustments were made based on the feedback received from five young adults (aged 20-39 who had not yet received the COVID-19 vaccine) who viewed the video and participated a focus group in June 2021."

6b) Any changes to trial outcomes after the trial commenced, with reasons

Does your paper address CONSORT subitem 6b? *
Copy and paste relevant sections from the manuscript (include quotes in quotation marks "like this" to indicate direct quotes from your manuscript), or elaborate on this item by providing additional information not in the ms, or briefly explain why the item is not applicable/relevant for your study

N/A - no change in trial outcomes

7a) How sample size was determined
NPT: When applicable, details of whether and how the clustering by care provides or centers was addressed

7a-i) Describe whether and how expected attrition was taken into account when calculating the sample size
Describe whether and how expected attrition was taken into account when calculating the sample size.

1 2 3 4 5

subitem not at all important ○ ○ ○ ● ○ essential

Clear selection

Does your paper address subitem 7a-i?

Copy and paste relevant sections from manuscript title (include quotes in quotation marks "like this" to indicate direct quotes from your manuscript), or elaborate on this item by providing additional information not in the ms, or briefly explain why the item is not applicable/relevant for your study

"To calculate the required sample size for the within-participant change in vaccine hesitancy (i.e., pre-post intervention) we used survey data that showed that in January/February 2021, approximately 40% of Canadians aged 20-39 were hesitant towards a COVID-19 vaccine i.e., don't know yet or would refuse vaccination[52, 53]. Estimating a 5% decrease of hesitancy in the intervention group and a correlation of about 0.4 between paired observations, the intervention group required a sample size of 907 pairs for detecting a 5% change of marginal proportions at a power of 80% and two-sided significance of 5% [54]. To detect a 5% superiority of the video intervention in increasing vaccine intentions compared to the active control group at a power of 80% we estimated a required sample per group of about 1300 participants. Considering a 1:1 allocation, the total sample required for this study was approximately 2600 participants (2*1300=2600)."

7b) When applicable, explanation of any interim analyses and stopping guidelines

Does your paper address CONSORT subitem 7b? *

Copy and paste relevant sections from the manuscript (include quotes in quotation marks "like this" to indicate direct quotes from your manuscript), or elaborate on this item by providing additional information not in the ms, or briefly explain why the item is not applicable/relevant for your study

"Data collection took place from July 30 to September 13, 2021. At about 5 weeks into data collection daily recruitment significantly declined. The main barrier was the relative low proportion (about 22%) of eligible participants (i.e., unvaccinated in the age group 20 to 39 years). We conducted preliminary analyses using a total sample of 1346 participants (673 per group) and found that the number of observations ensured 80% power to detect a 5% pre-post change in vaccine intentions. Preliminary analyses showed a difference of about 2% as opposed to the expected between-group difference of 5% in vaccine intentions which we had anticipated. To reach a similar level of power would have required about 5500 participants per group (i.e., an increase of 4200 from our initial sample calculations) to detect a statistically significant superiority of the video-intervention. Reaching the new sample size target would not have been feasible due to time and budget considerations and we decided to stop data collection."

8a) Method used to generate the random allocation sequence

NPT: When applicable, how care providers were allocated to each trial group

Does your paper address CONSORT subitem 8a? *

Copy and paste relevant sections from the manuscript (include quotes in quotation marks "like this" to indicate direct quotes from your manuscript), or elaborate on this item by providing additional information not in the ms, or briefly explain why the item is not applicable/relevant for your study

"Participants then viewed an online consent form, which informed them that they would be randomly assigned to watch a video or read a text related to the COVID-19 pandemic. After providing consent, participants deemed eligible to participate were randomly allocated to one of the 16 strata based on the four quota sampling criteria i.e., primary language, biological sex, income, and population density (See Appendix B for details). Within each stratum, a random concept picker approach was used to ensure a 1:1 allocation. Correspondingly, the first participant of a pair was randomly allocated to the intervention or the control arm and the second participant to the opposite arm. If a participant did not finish the survey (incomplete data), that place in the pair was allocated to the next participant. Thus, the quota in each stratum was filled in pairs and ensured a balanced group allocation throughout the data collection period."

8b) Type of randomisation; details of any restriction (such as blocking and block size)

Does your paper address CONSORT subitem 8b? *

Copy and paste relevant sections from the manuscript (include quotes in quotation marks "like this" to indicate direct quotes from your manuscript), or elaborate on this item by providing additional information not in the ms, or briefly explain why the item is not applicable/relevant for your study

"Within each stratum, a random concept picker approach was used to ensure a 1:1 allocation. Correspondingly, the first participant of a pair was randomly allocated to the intervention or the control arm and the second participant to the opposite arm. If a participant did not finish the survey (incomplete data), that place in the pair was allocated to the next participant. Thus, the quota in each stratum was filled in pairs and ensured a balanced group allocation throughout the data collection period. "

9) Mechanism used to implement the random allocation sequence (such as sequentially numbered containers), describing any steps taken to conceal the sequence until interventions were assigned

Does your paper address CONSORT subitem 9? *

Copy and paste relevant sections from the manuscript (include quotes in quotation marks "like this" to indicate direct quotes from your manuscript), or elaborate on this item by providing additional information not in the ms, or briefly explain why the item is not applicable/relevant for your study

"Within each stratum, a random concept picker approach was used to ensure a 1:1 allocation. Correspondingly, the first participant of a pair was randomly allocated to the intervention or the control arm and the second participant to the opposite arm. If a participant did not finish the survey (incomplete data), that place in the pair was allocated to the next participant. Thus, the quota in each stratum was filled in pairs and ensured a balanced group allocation throughout the data collection period."

10) Who generated the random allocation sequence, who enrolled participants, and who assigned participants to interventions

Does your paper address CONSORT subitem 10? *

Copy and paste relevant sections from the manuscript (include quotes in quotation marks "like this" to indicate direct quotes from your manuscript), or elaborate on this item by providing additional information not in the ms, or briefly explain why the item is not applicable/relevant for your study

"Data collection was carried out by Dynata, an international online market research company with experience in programing surveys and collecting data for universities and companies in various fields (e.g., public health, politics)."

11a) If done, who was blinded after assignment to interventions (for example, participants, care providers, those assessing outcomes) and how

NPT: Whether or not administering co-interventions were blinded to group assignment

11a-i) Specify who was blinded, and who wasn't

Specify who was blinded, and who wasn't. Usually, in web-based trials it is not possible to blind the participants [1, 3] (this should be clearly acknowledged), but it may be possible to blind outcome assessors, those doing data analysis or those administering co-interventions (if any).

1 2 3 4 5

subitem not at all important ○ ○ ○ ○ ● essential

Clear selection

Does your paper address subitem 11a-i? *

Copy and paste relevant sections from the manuscript (include quotes in quotation marks "like this" to indicate direct quotes from your manuscript), or elaborate on this item by providing additional information not in the ms, or briefly explain why the item is not applicable/relevant for your study

N/A - no blinding in study design

11a-ii) Discuss e.g., whether participants knew which intervention was the "intervention of interest" and which one was the "comparator"

Informed consent procedures (4a-ii) can create biases and certain expectations - discuss e.g., whether participants knew which intervention was the "intervention of interest" and which one was the "comparator".

1 2 3 4 5

subitem not at all important ○ ○ ○ ○ ● essential

Clear selection

Does your paper address subitem 11a-ii?

Copy and paste relevant sections from the manuscript (include quotes in quotation marks "like this" to indicate direct quotes from your manuscript), or elaborate on this item by providing additional information not in the ms, or briefly explain why the item is not applicable/relevant for your study

"Participants then viewed an electronic consent form, which informed them that they would be randomly assigned to watch a video or read a text related to the COVID-19 pandemic."

No information was provided to indicate which intervention was the "intervention of interest".

11b) If relevant, description of the similarity of interventions

(this item is usually not relevant for ehealth trials as it refers to similarity of a placebo or sham intervention to a active medication/intervention)

Does your paper address CONSORT subitem 11b? *

Copy and paste relevant sections from the manuscript (include quotes in quotation marks "like this" to indicate direct quotes from your manuscript), or elaborate on this item by providing additional information not in the ms, or briefly explain why the item is not applicable/relevant for your study

"The text-based intervention was limited to about 450 words to ensure a reading time similar to the duration of the video-based intervention"

12a) Statistical methods used to compare groups for primary and secondary outcomes

NPT: When applicable, details of whether and how the clustering by care providers or centers was addressed

Does your paper address CONSORT subitem 12a? *

Copy and paste relevant sections from the manuscript (include quotes in quotation marks "like this" to indicate direct quotes from your manuscript), or elaborate on this item by providing additional information not in the ms, or briefly explain why the item is not applicable/relevant for your study

" For baseline socio-demographics, we calculated proportions, means (and standard deviation, SD) and used the Pearson Chi-Square Test and the Welch two sample t-test to evaluate if the two study groups differed significantly. At baseline and post-intervention and for each of the study groups we calculated the proportion of participants in each of the four PAPM intention stage i.e., unengaged, undecided, decided not), and decided to. For each study group we calculated the pre-to post transitions in intentions to receive the COVID-19 vaccine. To estimate the pre-to-post intervention change in vaccine intentions we used a binary outcome (i.e., "Intenders" corresponding to the stage decided to; and "Non-intenders" that included stages unengaged, undecided, and decided not) and the McNemar's Chi-square test. To estimate pre to post changes in PAPM intention stages we conducted exact tests of symmetry (4x4 contingency tables) that comprise pairwise McNemar tests (using the "nominalSymmetryTest" function available in the R package "rcompanion") [56]. We reported adjusted p values for multiple comparisons (Benjamini & Hochberg method), odds ratios (OR) and Cohen's g effect size that was interpreted as small (0.05 to < 0.15), medium (0.15 to < 0.25) or large (≥ 0.25). For each study group we used the significant transitions between vaccine intention stage pairs for calculating the total number of participants that changed towards increased vaccination intentions (e.g., from undecided to decided to) and estimated the between-group difference using the Chi-square two-sample test for equality of proportions. To estimate the between-group difference in vaccine intentions, the Pearson Chi-Square Test was conducted on post-intervention vaccine intentions using the four-stage PAPM outcome. "

12a-i) Imputation techniques to deal with attrition / missing values

Imputation techniques to deal with attrition / missing values: Not all participants will use the intervention/comparator as intended and attrition is typically high in ehealth trials. Specify how participants who did not use the application or dropped out from the trial were treated in the statistical analysis (a complete case analysis is strongly discouraged, and simple imputation techniques such as LOCF may also be problematic [4]).

1 2 3 4 5

subitem not at all important ○ ● ○ ○ ○ essential

Clear selection

Does your paper address subitem 12a-i? *

Copy and paste relevant sections from the manuscript (include quotes in quotation marks "like this" to indicate direct quotes from your manuscript), or elaborate on this item by providing additional information not in the ms, or briefly explain why the item is not applicable/relevant for your study

N/A - no missing data as participants were not able to skip questions.

12b) Methods for additional analyses, such as subgroup analyses and adjusted analyses

Does your paper address CONSORT subitem 12b? *

Copy and paste relevant sections from the manuscript (include quotes in quotation marks "like this" to indicate direct quotes from your manuscript), or elaborate on this item by providing additional information not in the ms, or briefly explain why the item is not applicable/relevant for your study

" Additional analyses
Using the same analysis approach, we performed two subgroup analyses that included 1) all participants who answered the post-intervention COVID-19 vaccine intentions question and participants who were initially removed during data cleaning (n=1654); and 2) all participants who were randomly allocated to the study groups and who answered the pre-intervention COVID-19 vaccine intentions question (n=2089, intention to-treat approach). In addition, for both subgroups, we performed exploratory between-group analyses and operationalized the vaccine intention outcome in two different ways: 1) baseline (pre intervention) vaccine intentions in the text group and post intervention intentions in the video group; and 2) post intervention vaccine intentions in the text group and baseline intentions in the video group. "

X26) REB/IRB Approval and Ethical Considerations [recommended as subheading under "Methods"] (not a CONSORT item)

X26-i) Comment on ethics committee approval

1 2 3 4 5

subitem not at all important ○ ○ ○ ○ ● essential

Clear selection

Does your paper address subitem X26-i?

Copy and paste relevant sections from the manuscript (include quotes in quotation marks "like this" to indicate direct quotes from your manuscript), or elaborate on this item by providing additional information not in the ms, or briefly explain why the item is not applicable/relevant for your study

"Ethical considerations
The study was approved by the Research Ethics Board of the CIUSSS West-Central Montreal (Project ID #2021-2732)."

x26-ii) Outline informed consent procedures

Outline informed consent procedures e.g., if consent was obtained offline or online (how? Checkbox, etc.?), and what information was provided (see 4a-ii). See [6] for some items to be included in informed consent documents.

1 2 3 4 5

subitem not at all important ○ ○ ○ ● ○ essential

Clear selection

Does your paper address subitem X26-ii?

Copy and paste relevant sections from the manuscript (include quotes in quotation marks "like this" to indicate direct quotes from your manuscript), or elaborate on this item by providing additional information not in the ms, or briefly explain why the item is not applicable/relevant for your study

" Participants then viewed an online consent form, which informed them that they would be randomly assigned to watch a video or read a text related to the COVID-19 pandemic. "

X26-iii) Safety and security procedures

Safety and security procedures, incl. privacy considerations, and any steps taken to reduce the likelihood or detection of harm (e.g., education and training, availability of a hotline)

1 2 3 4 5

subitem not at all important ○ ● ○ ○ ○ essential

Clear selection

Does your paper address subitem X26-iii?

Copy and paste relevant sections from the manuscript (include quotes in quotation marks "like this" to indicate direct quotes from your manuscript), or elaborate on this item by providing additional information not in the ms, or briefly explain why the item is not applicable/relevant for your study

N/A - participants were provided with information about mental health resources and research complaints in the informed consent form, but there are no known harms of administering an altruism-based video intervention related to vaccination. Additionally, they were informed that data collection was anonymous.

RESULTS

13a) For each group, the numbers of participants who were randomly assigned, received intended treatment, and were analysed for the primary outcome

NPT: The number of care providers or centers performing the intervention in each group and the number of patients treated by each care provider in each center

Does your paper address CONSORT subitem 13a? *

Copy and paste relevant sections from the manuscript (include quotes in quotation marks "like this" to indicate direct quotes from your manuscript), or elaborate on this item by providing additional information not in the ms, or briefly explain why the item is not applicable/relevant for your study

"Out of 14298 participants in the target age group who accessed the invitation to participate, 11853 were assessed for eligibility, and 2097 were eligible (n = 9,578 were excluded because they were already vaccinated against COVID-19, n = 174 did not meet other inclusion criteria, n = 4 dropped out) and were randomly allocated to the study arms; 1654 completed the post intervention assessment and 1373 (i.e., 686 and 687 in the video and text intervention arms respectively) were included in the analyses. (See Figure 1, CONSORT diagram)."

13b) For each group, losses and exclusions after randomisation, together with reasons

Does your paper address CONSORT subitem 13b? (NOTE: Preferably, this is shown in a CONSORT flow diagram) *

Copy and paste relevant sections from the manuscript (include quotes in quotation marks "like this" to indicate direct quotes from your manuscript), or elaborate on this item by providing additional information not in the ms, or briefly explain why the item is not applicable/relevant for your study

"Out of 14298 participants in the target age group who accessed the invitation to participate, 11853 were assessed for eligibility, and 2097 were eligible (n = 9,578 were excluded because they were already vaccinated against COVID-19, n = 174 did not meet other inclusion criteria, n = 4 dropped out) and were randomly allocated to the study arms; 1654 completed the post intervention assessment and 1373 (i.e., 686 and 687 in the video and text intervention arms respectively) were included in the analyses. (See Figure 1, CONSORT diagram)."

13b-i) Attrition diagram

Strongly recommended: An attrition diagram (e.g., proportion of participants still logging in or using the intervention/comparator in each group plotted over time, similar to a survival curve) or other figures or tables demonstrating usage/dose/engagement.

1 2 3 4 5

subitem not at all important ○ ○ ○ ○ ○ essential

Does your paper address subitem 13b-i?

Copy and paste relevant sections from the manuscript or cite the figure number if applicable (include quotes in quotation marks "like this" to indicate direct quotes from your manuscript), or elaborate on this item by providing additional information not in the ms, or briefly explain why the item is not applicable/relevant for your study

Yes - See Figure 1, CONSORT diagram

14a) Dates defining the periods of recruitment and follow-up

Does your paper address CONSORT subitem 14a? *

Copy and paste relevant sections from the manuscript (include quotes in quotation marks "like this" to indicate direct quotes from your manuscript), or elaborate on this item by providing additional information not in the ms, or briefly explain why the item is not applicable/relevant for your study

"Data collection took place from July 30 to September 13, 2021."

14a-i) Indicate if critical "secular events" fell into the study period

Indicate if critical "secular events" fell into the study period, e.g., significant changes in Internet resources available or "changes in computer hardware or Internet delivery resources"

1 2 3 4 5

subitem not at all important ○ ○ ○ ○ ○ essential

Does your paper address subitem 14a-i?

Copy and paste relevant sections from the manuscript (include quotes in quotation marks "like this" to indicate direct quotes from your manuscript), or elaborate on this item by providing additional information not in the ms, or briefly explain why the item is not applicable/relevant for your study

N/A - no changes to internet resources during recruitment.

14b) Why the trial ended or was stopped (early)

Does your paper address CONSORT subitem 14b? *

Copy and paste relevant sections from the manuscript (include quotes in quotation marks "like this" to indicate direct quotes from your manuscript), or elaborate on this item by providing additional information not in the ms, or briefly explain why the item is not applicable/relevant for your study

"At about 5 weeks into data collection daily recruitment significantly declined. The main barrier was the relative low proportion (about 22%) of eligible participants (i.e., unvaccinated in the age group 20 to 39 years). We conducted preliminary analyses using a total sample of 1346 participants (673 per group) and found that the number of observations ensured 80% power to detect a 5% pre-post change in vaccine intentions. Preliminary analyses showed a difference of about 2% as opposed to the expected between-group difference of 5% in vaccine intentions which we had anticipated. To reach a similar level of power would have required about 5500 participants per group (i.e., an increase of 4200 from our initial sample calculations) to detect a statistically significant superiority of the video-intervention. Reaching the new sample size target would not have been feasible due to time and budget considerations and we decided to stop data collection."

15) A table showing baseline demographic and clinical characteristics for each group

NPT: When applicable, a description of care providers (case volume, qualification, expertise, etc.) and centers (volume) in each group

Does your paper address CONSORT subitem 15? *

Copy and paste relevant sections from the manuscript (include quotes in quotation marks "like this" to indicate direct quotes from your manuscript), or elaborate on this item by providing additional information not in the ms, or briefly explain why the item is not applicable/relevant for your study

"The sample consisted of slightly more females (53.9%); the mean age was 30.7 years; the majority used English as primary language at home (81.7%); most reported a total gross household income in the year preceding the pandemic of less than $75,000 Canadian (61.8%); and most resided in an urban area (77.7%). None of the sociodemographic characteristics differed significantly between the study groups (see Table 1 and Appendix E for additional subgroup analyses). In the video group 12.5% intended to receive the vaccine, 42.6% were decided against vaccination, 34.1% were undecided and 10.8% had not thought about receiving the COVID-19 vaccine (i.e., unengaged). Participants allocated to the active control group (text intervention) reported similar vaccine intentions and the difference between groups was not statistically significant: (χ^2(3) = 1.62, P = .65, see Table 2)."

See Table 1

15-i) Report demographics associated with digital divide issues

In ehealth trials it is particularly important to report demographics associated with digital divide issues, such as age, education, gender, social-economic status, computer/Internet/ehealth literacy of the participants, if known.

1 2 3 4 5

subitem not at all important ○ ○ ● ○ ○ essential

Clear selection

Does your paper address subitem 15-i? *

Copy and paste relevant sections from the manuscript (include quotes in quotation marks "like this" to indicate direct quotes from your manuscript), or elaborate on this item by providing additional information not in the ms, or briefly explain why the item is not applicable/relevant for your study

"The sample consisted of slightly more females (53.9%); the mean age was 30.7 years; the majority used English as primary language at home (81.7%); most reported a total gross household income in the year preceding the pandemic of less than $75,000 Canadian (61.8%); and most resided in an urban area (77.7%). None of the sociodemographic characteristics differed significantly between the study groups (see Table 1 and Appendix E for additional subgroup analyses). In the video group 12.5% intended to receive the vaccine, 42.6% were decided against vaccination, 34.1% were undecided and 10.8% had not thought about receiving the COVID-19 vaccine (i.e., unengaged). Participants allocated to the active control group (text intervention) reported similar vaccine intentions and the difference between groups was not statistically significant: (χ^2(3) = 1.62, P = .65, see Table 2)."

See Table 1

16) For each group, number of participants (denominator) included in each analysis and whether the analysis was by original assigned groups

16-i) Report multiple "denominators" and provide definitions

Report multiple "denominators" and provide definitions: Report N's (and effect sizes) "across a range of study participation [and use] thresholds" [1], e.g., N exposed, N consented, N used more than x times, N used more than y weeks, N participants "used" the intervention/comparator at specific pre-defined time points of interest (in absolute and relative numbers per group). Always clearly define "use" of the intervention.

1 2 3 4 5

subitem not at all important ○ ○ ○ ○ ● essential

Clear selection

Does your paper address subitem 16-i? *

Copy and paste relevant sections from the manuscript (include quotes in quotation marks "like this" to indicate direct quotes from your manuscript), or elaborate on this item by providing additional information not in the ms, or briefly explain why the item is not applicable/relevant for your study

"1373 (i.e., 686 and 687 in the video and text intervention arms respectively) were included in the analyses. (See Figure 1, CONSORT diagram)."

16-ii) Primary analysis should be intent-to-treat

Primary analysis should be intent-to-treat, secondary analyses could include comparing only "users", with the appropriate caveats that this is no longer a randomized sample (see 18-i).

1 2 3 4 5

subitem not at all important ○ ○ ● ○ ○ essential

Clear selection

Does your paper address subitem 16-ii?

Copy and paste relevant sections from the manuscript (include quotes in quotation marks "like this" to indicate direct quotes from your manuscript), or elaborate on this item by providing additional information not in the ms, or briefly explain why the item is not applicable/relevant for your study

"Using the same analysis approach, we performed two subgroup analyses that included 1) all participants who answered the post-intervention COVID-19 vaccine intentions question and participants who were initially removed during data cleaning (n=1654); and 2) all participants who were randomly allocated to the study groups and who answered the pre-intervention COVID-19 vaccine intentions question (n=2089, intention to-treat approach)."

"Results of additional subgroup analyses did not significantly differ from per protocol analyses (see Appendix D)"

17a) For each primary and secondary outcome, results for each group, and the estimated effect size and its precision (such as 95% confidence interval)

Does your paper address CONSORT subitem 17a? *

Copy and paste relevant sections from the manuscript (include quotes in quotation marks "like this" to indicate direct quotes from your manuscript), or elaborate on this item by providing additional information not in the ms, or briefly explain why the item is not applicable/relevant for your study

Yes - See Table 2 and Figures 1 & 2

17a-i) Presentation of process outcomes such as metrics of use and intensity of use

In addition to primary/secondary (clinical) outcomes, the presentation of process outcomes such as metrics of use and intensity of use (dose, exposure) and their operational definitions is critical. This does not only refer to metrics of attrition (13-b) (often a binary variable), but also to more continuous exposure metrics such as "average session length". These must be accompanied by a technical description how a metric like a "session" is defined (e.g., timeout after idle time) [1] (report under item 6a).

1 2 3 4 5

subitem not at all important ○ ● ○ ○ ○ essential

Clear selection

Does your paper address subitem 17a-i?

Copy and paste relevant sections from the manuscript (include quotes in quotation marks "like this" to indicate direct quotes from your manuscript), or elaborate on this item by providing additional information not in the ms, or briefly explain why the item is not applicable/relevant for your study

N/A - cross-sectional web-based survey

17b) For binary outcomes, presentation of both absolute and relative effect sizes is recommended

Does your paper address CONSORT subitem 17b? *

Copy and paste relevant sections from the manuscript (include quotes in quotation marks "like this" to indicate direct quotes from your manuscript), or elaborate on this item by providing additional information not in the ms, or briefly explain why the item is not applicable/relevant for your study

"In the video group, 43 participants changed from non-intenders at baseline (i.e., unengaged, undecided, or decided not) to vaccine intenders (i.e., decided to) post intervention and 10 participants changed from intenders at baseline to non-intenders post intervention. The McNemar test was significant (χ^2(1) = 20.55, P < .001). In the active control (text) group, 24 participants changed from non-intenders at baseline to vaccine intenders post intervention and 5 participants changed from intenders to non-intenders. Unexpectedly, the McNemar test was also significant (χ^2(1) = 12.45, P < .001)."

18) Results of any other analyses performed, including subgroup analyses and adjusted analyses, distinguishing pre-specified from exploratory

Does your paper address CONSORT subitem 18? *

Copy and paste relevant sections from the manuscript (include quotes in quotation marks "like this" to indicate direct quotes from your manuscript), or elaborate on this item by providing additional information not in the ms, or briefly explain why the item is not applicable/relevant for your study

"Results of additional subgroup analyses did not significantly differ from per protocol analyses (see Appendix D). The only difference consists in the loss of statistical significance of the transition from unengaged to decided to in the video group (see Table 2) that can be explained by two additional participants who transitioned from decided to at baseline to unengaged post intervention. Since one cannot change from decided to get the vaccine to unengaged, this is an artifact introduced by careless responding.
Results of exploratory analyses provide a signal that the video intervention was superior to the text intervention as the between-group difference in vaccine intentions was significant when using pre-intervention intentions in the text group and post intervention intentions in the video group (χ^2(1) = 5.90, P = .02) and not significant when using pre-intervention intentions in the video group and post intervention intentions in the text group (χ^2(1) = 2.39, P = .12) (see Appendix D, Tables 10 and 11). The same results were obtained using the n = 1654 (all completers of the second vaccination intention assessment, see Appendix D, Tables 5 and 6) and n = 2089 (ITT) samples (see Appendix D, Tables 8 and 9)."

18-i) Subgroup analysis of comparing only users

A subgroup analysis of comparing only users is not uncommon in ehealth trials, but if done, it must be stressed that this is a self-selected sample and no longer an unbiased sample from a randomized trial (see 16-iii).

subitem not at all important 1 2 (●) 3 4 5 essential

Clear selection

Does your paper address subitem 18-i?

Copy and paste relevant sections from the manuscript (include quotes in quotation marks "like this" to indicate direct quotes from your manuscript), or elaborate on this item by providing additional information not in the ms, or briefly explain why the item is not applicable/relevant for your study

N/A - sample was not self-selected

19) All important harms or unintended effects in each group

(for specific guidance see CONSORT for harms)

Does your paper address CONSORT subitem 19? *

Copy and paste relevant sections from the manuscript (include quotes in quotation marks "like this" to indicate direct quotes from your manuscript), or elaborate on this item by providing additional information not in the ms, or briefly explain why the item is not applicable/relevant for your study

N/A - there are no known harms of watching an altruism-based video related to vaccination or reading a text on preventive health behaviours, and no adverse events were reported.

19-i) Include privacy breaches, technical problems

Include privacy breaches, technical problems. This does not only include physical "harm" to participants, but also incidents such as perceived or real privacy breaches [1], technical problems, and other unexpected/unintended incidents. "Unintended effects" also includes unintended positive effects [2].

subitem not at all important 1 2 3 (●) 4 5 essential

Clear selection

Does your paper address subitem 19-i?

Copy and paste relevant sections from the manuscript (include quotes in quotation marks "like this" to indicate direct quotes from your manuscript), or elaborate on this item by providing additional information not in the ms, or briefly explain why the item is not applicable/relevant for your study

N/A - As Dynata sends researchers anonymized data, we cannot identify participants other than through reported gender, sex, and province of residence

19-ii) Include qualitative feedback from participants or observations from staff/researchers

Include qualitative feedback from participants or observations from staff/researchers, if available, on strengths and shortcomings of the application, especially if they point to unintended/unexpected effects or uses. This includes (if available) reasons for why people did or did not use the application as intended by the developers.

subitem not at all important 1 2 3 (●) 4 5 essential

Clear selection

Does your paper address subitem 19-ii?

Copy and paste relevant sections from the manuscript (include quotes in quotation marks "like this" to indicate direct quotes from your manuscript), or elaborate on this item by providing additional information not in the ms, or briefly explain why the item is not applicable/relevant for your study

No qualitative feedback was obtained from the participants who completed the survey, but focus groups were used to refine the video intervention before the trial began. Additionally research collaborators (ED, GZ, MT) outside of the internal research team were shown the video and asked to provide feedback on its appropriateness to COVID-19 vaccination.

"Adjustments were made based on the feedback received from five young adults (aged 20-39 who had not yet received the COVID-19 vaccine) who viewed the video and participated a focus group in June 2021."

DISCUSSION

22) Interpretation consistent with results, balancing benefits and harms, and considering other relevant evidence

NPT: In addition, take into account the choice of the comparator, lack of or partial blinding, and unequal expertise of care providers or centers in each group

22-i) Restate study questions and summarize the answers suggested by the data, starting with primary outcomes and process outcomes (use)

Restate study questions and summarize the answers suggested by the data, starting with primary outcomes and process outcomes (use).

subitem not at all important 1 2 3 4 (●) 5 essential

Clear selection

Does your paper address subitem 22-i? *

Copy and paste relevant sections from the manuscript (include quotes in quotation marks "like this" to indicate direct quotes from your manuscript), or elaborate on this item by providing additional information not in the ms, or briefly explain why the item is not applicable/relevant for your study

"To our knowledge, this is the first population-based study that examined the effect of a video-based intervention eliciting prosocial (altruistic) motives on intentions to receive the COVID-19 vaccine in younger Canadian adults. We used a pre- post and RCT study design and recruited a national sample of unvaccinated 20 to 39 years old Canadians who participated in a web-based survey between July-September 2021, in the context of the fourth COVID-19 pandemic wave. Our study had two specific objectives: (1) to estimate pre- to post intervention vaccine intention changes in participants who were randomly allocated to the video intervention or the text-based intervention that provided non-vaccine related preventive health measures, and (2) to estimate between-group vaccine intentions post-intervention."

Results are then discussed in detail.

22-ii) Highlight unanswered new questions, suggest future research

Highlight unanswered new questions, suggest future research.

subitem not at all important 1 2 3 4 (●) 5 essential

Clear selection

Does your paper address subitem 22-ii?

Copy and paste relevant sections from the manuscript (include quotes in quotation marks "like this" to indicate direct quotes from your manuscript), or elaborate on this item by providing additional information not in the ms, or briefly explain why the item is not applicable/relevant for your study

"While achieving statistical significance for the between group difference would have sent a strong signal related to the efficacy of the video intervention, we believe that our study can inform future research using interventions that elicit prosocial motives to increase COVID-19 vaccine intentions. For example, interventions could be adapted to include other forms of prosocial motivations such as collectivism (the practice of prioritizing a group over individuals within the group) [63]."

"follow-up 3-6 months later would have allowed us to evaluate the translation of increased vaccine intentions into actual vaccine uptake."

20) Trial limitations, addressing sources of potential bias, imprecision, and, if relevant, multiplicity of analyses

20-i) Typical limitations in ehealth trials

Typical limitations in ehealth trials: Participants in ehealth trials are rarely blinded. Ehealth trials often look at a multiplicity of outcomes, increasing risk for a Type I error. Discuss biases due to non-use of the intervention/usability issues, biases through informed consent procedures, unexpected events.

subitem not at all important 1 2 3 (●) 4 5 essential

Clear selection

Does your paper address subitem 20-i? *

Copy and paste relevant sections from the manuscript (include quotes in quotation marks "like this" to indicate direct quotes from your manuscript), or elaborate on this item by providing additional information not in the ms, or briefly explain why the item is not applicable/relevant for your study

"The main limitations derive from the premature termination of the study dictated by barriers in participant recruitment and by lower-than-anticipated COVID-19 vaccine hesitancy in the population of interest. As the target sample size was not reached, the sampling quotas used to match Canadian Census data deviated from the planned quotas and we included 3.9% more females, 2.3% more participants residing in rural areas, 5.2% less Francophones and 11.8% less participants with annual total income before taxes of all members of the household before the pandemic of 75,000 CAD . While between-group differences were not significant, these differences in socio-demographics could impede the generalizability of the results to the Canadian population. The high proportion of participants who were in the decided not to vaccinate stage could have diminished our ability to prove the superiority of the video intervention in increasing vaccine intentions. Additionally, the use of an active control group could have diminished our capacity to prove the statistical superiority of the video intervention, perhaps due to social desirability. Finally, follow-up 3-6 months later would have allowed us to evaluate the translation of increased vaccine intentions into actual vaccine uptake."

21) Generalisability (external validity, applicability) of the trial findings

NPT: External validity of the trial findings according to the intervention, comparators, patients, and care providers or centers involved in the trial

21-i) Generalizability to other populations

Generalizability to other populations: In particular, discuss generalizability to a general Internet population, outside of a RCT setting, and general patient population, including applicability of the study results for other organizations

subitem not at all important 1 2 3 4 (●) 5 essential

Clear selection

Does your paper address subitem 21-i?

Copy and paste relevant sections from the manuscript (include quotes in quotation marks "like this" to indicate direct quotes from your manuscript), or elaborate on this item by providing additional information not in the ms, or briefly explain why the item is not applicable/relevant for your study

"The main limitations derive from the premature termination of the study dictated by barriers in participant recruitment and by lower-than-anticipated COVID-19 vaccine hesitancy in the population of interest. As the target sample size was not reached, the sampling quotas used to match Canadian Census data deviated from the planned quotas and we included 3.9% more females, 2.3% more participants residing in rural areas, 5.2% less Francophones and 11.8% less participants with annual total income before taxes of all members of the household before the pandemic of 75,000 CAD . While between-group differences were not significant, these differences in socio-demographics could impede the generalizability of the results to the Canadian population."

21-ii) Discuss if there were elements in the RCT that would be different in a routine application setting

Discuss if there were elements in the RCT that would be different in a routine application setting (e.g., prompts/reminders, more human involvement, training sessions or other co-interventions) and what impact the omission of these elements could have on use, adoption, or outcomes if the intervention is applied outside of a RCT setting.

subitem not at all important 1 2 3 4 5 essential

Does your paper address subitem 21-ii?

Copy and paste relevant sections from the manuscript (include quotes in quotation marks "like this" to indicate direct quotes from your manuscript), or elaborate on this item by providing additional information not in the ms, or briefly explain why the item is not applicable/relevant for your study

N/A - the video would still be viewed the same way in a routine application setting

OTHER INFORMATION

23) Registration number and name of trial registry

Does your paper address CONSORT subitem 23? *

Copy and paste relevant sections from the manuscript (include quotes in quotation marks "like this" to indicate direct quotes from your manuscript), or elaborate on this item by providing additional information not in the ms, or briefly explain why the item is not applicable/relevant for your study

"Trial Registration
This study was registered on ClinicalTrials.gov (NCT04960228)."

24) Where the full trial protocol can be accessed, if available

Does your paper address CONSORT subitem 24? *

Cite a Multimedia Appendix, other reference, or copy and paste relevant sections from the manuscript (include quotes in quotation marks "like this" to indicate direct quotes from your manuscript), or elaborate on this item by providing additional information not in the ms, or briefly explain why the item is not applicable/relevant for your study

N/A - no trial protocol is published.

25) Sources of funding and other support (such as supply of drugs), role of funders

Does your paper address CONSORT subitem 25? *

Copy and paste relevant sections from the manuscript (include quotes in quotation marks "like this" to indicate direct quotes from your manuscript), or elaborate on this item by providing additional information not in the ms, or briefly explain why the item is not applicable/relevant for your study

"This study was supported by a grant from the McGill Interdisciplinary Initiative in Infection and Immunity (MI4). OT is supported by the Canadian Institutes of Health Research (CIHR)-Frederick Banting and Charles Best Doctoral award (Award No. FBD-170837) outside the submitted work."

X27) Conflicts of Interest (not a CONSORT item)

X27-i) State the relation of the study team towards the system being evaluated

In addition to the usual declaration of interests (financial or otherwise), also state the relation of the study team towards the system being evaluated, i.e., state if the authors/evaluators are distinct from or identical with the developers/sponsors of the intervention.

subitem not at all important 1 2 3 4 (●) 5 essential

Clear selection

Does your paper address subitem X27-i?

Copy and paste relevant sections from the manuscript (include quotes in quotation marks "like this" to indicate direct quotes from your manuscript), or elaborate on this item by providing additional information not in the ms, or briefly explain why the item is not applicable/relevant for your study

"Within the last 5 years, Gregory Zimet has served as a consultant to Merck (regarding HPV vaccination), Pfizer (regarding meningococcal vaccination), and Sanofi Pasteur (regarding adolescent vaccination). Gregory Zimet also has served on advisory boards for Merck (regarding HPV vaccination) and Moderna (regarding COVID-19 vaccination)."

About the CONSORT EHEALTH checklist

As a result of using this checklist, did you make changes in your manuscript? *

- ○ yes, major changes
- ○ yes, minor changes
- ● no

What were the most important changes you made as a result of using this checklist?

Your answer

How much time did you spend on going through the checklist INCLUDING making changes in your manuscript *

6 hours were spent completing this checklist.

As a result of using this checklist, do you think your manuscript has improved? *

- ○ yes
- ● no
- ○ Other:

Would you like to become involved in the CONSORT EHEALTH group?

This would involve for example becoming involved in participating in a workshop and writing an "Explanation and Elaboration" document

- ○ yes
- ● ~~no~~
- ○ Other:

Clear selection

Any other comments or questions on CONSORT EHEALTH

Your answer

STOP - Save this form as PDF before you click submit

To generate a record that you filled in this form, we recommend to generate a PDF of this page (on a Mac, simply select "print" and then select "print as PDF") before you submit it.

When you submit your (revised) paper to JMIR, please upload the PDF as supplementary file.

Don't worry if some text in the textboxes is cut off, as we still have the complete information in our database. Thank you!

Final step: Click submit !

Click submit so we have your answers in our database!

Submit   Clear form

Never submit passwords through Google Forms.

This content is neither created nor endorsed by Google. Report Abuse - Terms of Service - Privacy Policy

Google Forms
